# Supplementary material for: Socioeconomic and behavioral determinants of cardiovascular diseases among older adults in Belgium and France: A longitudinal analysis from the SHARE study
Source: PLoS One. 2020 Dec 4;15(12):e0243422. doi: 10.1371/journal.pone.0243422 (PMC7717541; doi:10.1371/journal.pone.0243422)
Supplement: S1 Annex — The supplementary material contains detailed information on comparison of included and excluded participants, behavioral risks overtime, sensitivity analysis of multiple imputation and complete case analysis, checking for imputation model fit, comparison of models with various correlation structure, and stratified GEE analysis for Belgium and France. (PDF) [file pone.0243422.s001.pdf]

Socioeconomic and behavioral determinants of cardiovascular diseases among older adults in Belgium and France: a longitudinal analysis from the SHARE study

Hamid Yimam Hassen[1]*, Hilde Bastiaens[1,2], Kathleen Van Royen[1,4], Steven Abrams[2,3]

[1]Department of Primary and Interdisciplinary Care, Faculty of Medicine and Health Sciences, University of Antwerp, Antwerp, Belgium

[2]Global Health Institute, Faculty of Medicine and Health Sciences, University of Antwerp, Antwerp, Belgium

[3]Interuniversity Institute for Biostatistics and statistical Bioinformatics, Data Science Institute, Hasselt University, Diepenbeek, Belgium

[4]Department of Communication Studies, Faculty of Social Sciences, University of Antwerp, Antwerp, Belgium

*Corresponding author

Email: Hamid.Hassen@uantwerpen.be (HYH)

This supplementary material contains detailed information on comparison of included and excluded participants, trends of behavioral risks overtime, sensitivity analysis of multiple imputation and complete case analysis, checking for imputation model fit, comparison of models with various correlation structure, and stratified GEE analysis for Belgium and France.

Contents

Contents 2
Comparison of included and excluded participants 3
Trend of behavioral, physical measurements and clinical characteristics 3
Comparison of models using different correlation structure 5
Multiple imputation procedure 6
Imputation model fit 9
Stratified analysis for Belgium and France 10
References 11

Comparison of included and excluded participants

To check whether excluding individuals with no outcome information at enrollment biased the results, we compared socioeconomic characteristics between individuals included in the analysis (n = 14,322) and those excluded as a result of missing CVD outcome information at enrollment (n = 117), using two sample t-tests and chi-square tests for numerical and categorical variables, respectively. As shown in table 1, individuals who were included were comparable with the excluded ones in terms of socioeconomic characteristics, indicating the impact of missingness on the inference is very minimal.

Table 1. Comparison of the socioeconomic characteristics of participants included and excluded in the analysis among adults aged 50 or older in Belgium and France, 2011 to 2017 (*included=14,322; excluded due to missingness of outcome data at enrollment=117*)

Socioeconomic characteristics	Included (n=14,322; 96.8%)	Excluded (n=117; 0.8%)	P-value¥
**Age** (*mean/SD*)	64.9 (10.9)	65.3 (10.4)	0.618
**Sex** (female) n (%)	7975 (55.7)	64 (54.7)	0.829
**Living area** n (%)			
Rural	4965 (36.0)	41 (35.1)	0.975
Small	4263 (30.9)	37 (31.6)	
Large city/town	4565 (33.1)	39 (33.3)	
**Level of education** n (%)			
Primary	5902 (41.8)	46 (39.3)	0.937
Secondary	4327 (30.6)	34 (29.1)	
Higher	3901 (27.6)	37 (31.6)	
**Living with** (with partner) n (%)	9547 (67.0)	73 (64.6)	0.586
**Net income** (median/IQR)	30463.2 (31030.7)	29112.4 (30,170.6)	0.446
**Social support** (yes) n (%)	3589 (31.8)	37 (33.9)	0.633
**Retirement** (yes) n (%)	7939 (56.7)	69 (61.6)	0.302
**Family size**			
One	3869 (27.0)	31 (26.5)	0.171
Two	7989 (55.8)	63 (53.8)	
Three	1493 (10.4)	10 (8.5)	
Four and above	971 (6.8)	14 (12.0)	

¥ - P - values are based on two sample t-test and Chi-square test for numerical and categorical variables respectively.

Trend of behavioral, physical measurements and clinical characteristics

Table 2 shows the trend of behavioral, physical and other health related characteristics of participants at each wave of data collection.

Table 2. Behavioral, clinical, and physical characteristics of adults aged 50 years or older in Belgium and France (n=36,860 person observation points), from the Survey of Health, Ageing and Retirement in Europe, 2011 to 2017.

Behavioral and physical characteristics	Overall	2011	2013	2015	2017
**Fruit and vegetable** (n=23,641)					
Adequate [a]	20712 (87.6)	5934 (87.9)	5895 (88.4)	3348 (87.3)	5535 (86.7)
Not adequate	2929 (12.4)	815 (12.1)	775 (11.6)	487 (12.7)	852 (13.3)
**Vigorous physical activity** (n=31,473)					
More than once a week	8997 (28.6)	2352 (29.0)	2401 (27.4)	1835 (27.1)	2409 (30.8)
Once a week	4089 (13.0)	1036 (12.8)	1099 (12.6)	865 (12.8)	1089 (13.9)
One to three times a month	2369 (7.5)	626 (7.7)	663 (7.6)	508 (7.5)	572 (7.3)
Hardly ever, or never	16018 (50.9)	4109 (50.6)	4586 (52.4)	3560 (52.6)	3763 (48.0)
**Moderate physical activity** (n=31,471)					
More than once a week	20656 (65.6)	5386 (66.1)	5669 (64.8)	4296 (63.5)	5323 (68.0)
Once a week	4957 (15.8)	1270 (15.6)	1355 (15.5)	1073 (15.9)	1259 (16.1)
One to three times a month	1835 (5.8)	482 (5.9)	507 (5.8)	426 (6.3)	420 (5.4)
Hardly ever, or never	4023 (12.8)	1004 (12.4)	1215 (13.9)	973 (14.4)	831 (10.6)
**Regular alcohol consumption**[b] (n=11,928)					
Yes	932 (7.8)	130 (6.8)	183 (7.0)	342 (8.2)	277 (8.6)
No	10996 (92.2)	1780 (93.2)	2425 (93.0)	3831 (91.8)	2960 (91.4)
**Currently smoking** (n=26,825)					
Yes	5340 (19.9)	1605 (17.8)	1760 (17.7)	840 (21.3)	1135 (29.2)
No	21485 (80.1)	7430 (82.2)	8194 (82.3)	3108 (78.7)	2753 (70.8)
**Body Mass Index** (n=35,842)					
Normal	14912 (41.6)	3789 (42.9)	4061 (41.9)	3797 (40.6)	3265 (40.9)
Overweight	13817 (38.5)	3406 (38.6)	3697 (38.2)	3612 (38.6)	3102 (38.9)
Obesity	7113 (19.8)	1640 (18.6)	1923 (19.9)	1937 (20.7)	1613 (20.2)
**Depression** (n=30,587)					
Yes	9456 (30.9)	2681 (30.1)	3073 (31.4)	2915 (31.5)	787 (29.9)
No	21131 (69.1)	6232 (69.9)	6719 (68.6)	6339 (68.5)	1841 (70.1)
**Grip strength** *(mean)*	33.6 (11.8)	34.2 (12.0)	33.3 (11.9)	33.5 (11.7)	33.3 (11.6)
**Self-perceived health** (n=36,786)					
Excellent	2545 (6.9)	747 (8.2)	614 (6.2)	581 (6.1)	603 (7.4)
Very good	6596 (17.9)	1753 (19.2)	1625 (16.3)	1632 (17.0)	1586 (19.5)
Good	16041 (43.6)	4104 (45.0)	4261 (41.8)	4160 (43.4)	3516 (43.2)
Fair	8624 (23.4)	1907 (20.9)	2520 (25.3)	2355 (24.6)	1842 (22.6)
Poor	2980 (8.1)	615 (6.7)	925 (9.3)	849 (8.9)	591 (7.3)
**Number of co-morbid diseases**[c] (n=36,757)					
No comorbidity	14007 (38.1)	3718 (40.7)	3768 (37.9)	3622 (37.8)	2899 (35.8)
One comorbidity	11567 (31.5)	3029 (33.2)	3118 (31.4)	2945 (30.8)	2475 (30.5)
Two or more morbidity	11183 (30.4)	2383 (26.1)	3059 (30.8)	3010 (31.4)	2731 (33.7)

a- At least one serving of fruit and vegetable per day
b- Six or more units of alcoholic beverages on one occasion at least once a week
*c- **co-morbid diseases:** Chronic lung diseases, chronic kidney diseases, cancer, stomach and duodenal ulcer, Parkinson diseases, cataracts, rheumatoid arthritis, Alzheimer's disease, osteoarthritis*

Comparison of models using different correlation structure

Table 3. Comparison of multivariable GEE models of socioeconomic determinants of CVD among adults aged 50 or older in Belgium and France using different correlation structure. (*The model with exchangeable working correlation provides lowest QIC – 12,317.8*)

**Variables**	**Multivariable GEE analysis AOR [95%CI]**			
	**Exchangeable**	**Unstructured**	**Autoregressive**	**Independent**
**Age** (*cont.*)	1.057 [1.055–1.060]***	1.0557 [1.056–1.0565]***	1.057 [1.0566–1.0574]***	1.052 [1.051–1.053]***
**Sex**				
Male	1	1	1	1
Female	0.537 [0.511–0.564]***	0.541 [0.539–0.543]***	0.538 [0.536–0.539]***	0.548 [0.547–0.549]***
**Living area**				
Rural	1	1	1	1
Small	1.084 [0.995–1.137]	1.088 [0.979–1.210]	1.084 [0.985–1.194]	1.097 [0.999–1.204]
Large city/town	1.142 [1.069–1.182]*	1.164 [1.149–1.181]*	1.125 [1.096–1.154]*	1.114 [1.057–1.175]*
**Level of education**				
Primary	1	1	1	1
Secondary	0.911 [0.860–0.966]*	0.880 [0.851–0.910]*	0.895 [0.844–0.949]*	0.886 [0.851–0.923]*
Higher	0.824 [0.790–0.900]**	0.790 [0.779–0.802]**	0.828 [0.817–0.838]**	0.827 [0.819–0.836]**
**Marital status**				
Partner	1	1	1	1
Alone	1.045 [0.994–1.100]	1.061 [0.812–1.384]	1.050 [0.746–1.478]	1.047 [0.710–1.545]
**Net income**				
Lower	1	1		1
Middle	0.987 [0.945–1.031]	1.011 [0.463–2.206]	1.002 [0.457–2.198]	0.987 [0.492–1.981]
Upper	0.816 [0.756–0.857]**	0.897 [0.825–0.974]**	0.871 [0.845–0.897]**	0.810 [0.792–0.828]**
**Social support**				
No	1	1	1	1
Yes	0.805 [0.775–0.842]***	0.866 [0.844–0.889]***	0.860 [0.839–0.882]***	0.791 [0.769–0.812]***
**Retirement**				
No	1	1	1	1
Yes	1.211 [1.156–1.313]**	1.132 [1.104–1.160]**	1.110 [1.068–1.154]**	1.137 [1.106–1.169]**
Model comparisons				
**QIC**	12,317.8	12319.0	12318.4	12321.7
**Deviance**	12,295.92	12,290.32	12,292.88	12,288.52

QIC: quasi-likelihood under the Independence Model Criterion; AOR: Adjusted odds ratio

Multiple imputation procedure

There were missing values for level of education (1.3%), living condition (0.5%), living area (3.7%), income (1.5%), social support (21.2%), retirement (2.3%), fruit and vegetable intake (35.8%), physical activity level (14.6%), alcohol consumption (67.6%), smoking (27.2%), body mass index (2.8%), and depression (17.0%). We imputed the dataset to have M = 100 complete datasets using auxiliary variables in combination with the covariate information used in the substantive model(s) in order to specify the imputation models. Auxiliary variables used include family size, activities of daily living, limitation of activities, self-perceived health, current job situation, and the CASP-19 quality of life index. In general, the inclusion of auxiliary variables in the imputation models, next to the covariates presented in the substantive model(s), albeit potentially unimportant from an explanatory perspective, led to more precision in terms of estimation of the model parameters in the final substantive model. The perspective taken in the Multiple Imputation by Chained Equations (MICE) approach, also referred to as full conditional specification, is one of a specification of several imputation models which sequentially imputed missing values for a given variable when regressed against all other covariates available. Then, the parameters of interest were estimated in each imputed dataset separately, and combined using Rubin's rules (Rubin 2009). For more details regarding this approach and the justification of full conditional specification, we refer readers to a book by (Van Buuren 2018). The results of our sensitivity analysis are summarized in table 4 and 5 below.

Table 4. Sensitivity analysis of multivariable GEE models estimating the effect of socioeconomic characteristics on CVD among adults aged 50 or older in Belgium and France using multiple imputation and complete case analysis. The MI results provides more precise estimate and are reported in the main manuscript.

Variables	Multivariable GEE analysis AOR [95%CI]	
	**Multiple imputation**	**Complete case analysis**
**Age** (*cont.*)	1.057 [1.055–1.060]***	1.055 [1.048–1.061]***
**Sex**		
Male	1	1
Female	0.537 [0.511–0.564]***	0.592 [0.525–0.668]***
**Living area**		
Rural	1	1
Small	1.084 [0.995–1.137]	1.102 [0.966–1.258]
Large city/town	1.142 [1.069–1.182]*	1.174 [1.025–1.346]*
**Level of education**		
Primary	1	1
Secondary	0.911 [0.860–0.966]*	0.895 [0.777–1.031]
Higher	0.824 [0.790–0.900]**	0.825 [0.707–0.963]*
**Marital status**		
Partner	1	1
Alone	1.045 [0.994–1.100]	0.894 [0.787–1.014]
**Annual net income**		
Lower	1	1
Middle	0.987 [0.945–1.031]	0.953 [0.839–1.083]
Upper	0.816 [0.756–0.857]**	0.769 [0.661–0.894]***
**Social support**		
No	1	1
Yes	0.805 [0.775–0.842]***	0.781 [0.689–0.886]***
**Retirement**		
No	1	1
Yes	1.211 [1.156–1.313]**	1.159 [1.004–1.338]*
**Model comparisons**		
QIC	12,317.8	12,501.8
Wald $\chi2$ (d.f.)	2,582.66 (11)***	2,454.24 (11)***
Deviance	12,295.92	12,274.14

** p–value<0.05; ** p<0.01; *** p <0.001*

AOR: Adjusted odds ratio; COR: Crude dds ratio; MI: multiple imputations; CCA: complete case analysis; QIC: quasi-likelihood under the Independence Model Criterion

Multivariate multiple imputations were performed (n=36,860). We imputed 100 datasets

- *Interaction of age and level of education with living area, income and social support was assessed but statistically not significant.*

Table 5. Sensitivity analysis of multivariable GEE models estimating the effect of behavioral and physical determinants on cardiovascular diseases among adults aged 50 or older in Belgium and France using multiple imputation and complete case analysis. The MI results provides more precise estimate and are reported in the main manuscript.

Variables	Multivariable GEE analysis [AOR [95%CI]]	
	**Multiple imputation**	**Complete case analysis**
**Age** (*cont.*)	1.045 [1.042–1.048]***	1.039 [1.017–1.061]***
**Sex** *(female)*	0.418 [0.392–0.446]***	0.361 [0.210–0.620]***
**Physical activity**		
No regular PA	1	1
Regular PA	0.687 [0.639–0.734]**	0.576 [0.369–0.897]*
**Fruit and vegetable intake**		
Not adequate	1	1
Adequate	0.932 [0.873–0.996]*	0.887 [0.516–1.525]
**Smoking**		
No	1	1
Yes	1.190 [1.128–1.252]*	1.373 [0.894–2.107]
**Regular alcohol**		
No	1	1
Yes	1.042 [0.960–1.131]	0.784 [0.393–1.567]
**BMI**		
Normal	1	1
Overweight	1.082 [1.029–1.137]*	1.049 [0.686–1.604]
Obesity	1.495 [1.436–1.554]***	1.633 [1.024–2.605]*
**Chronic comorbidities**		
No comorbidity	1	1
One comorbidity	2.231 [2.100–2.373]***	3.362 [1.758–6.427]***
>=2 comorbidities	4.541 [4.269–4.831]***	7.721 [4.090–14.577]***
**Grip strength** (*cont.*)	0.987 [0.984–0.990]***	0.966 [0.941–0.992]***
**Depression**		
No		
Yes	1.269 [1.212–1.328]**	1.066 [0.724–1.570]
**Model comparisons**		
QIC	12,317.8	12,612.8
Wald χ2 (d.f.)	2,582.66 (12)***	2,143.24 (12)***
Deviance	12,295.92	12,187.14

** p–value<0.05; ** p<0.01; *** p <0.001*
AOR: Adjusted odds ratio; COR: Crude odds ratio; MI: multiple imputations; CCA: complete case analysis; QIC: quasi-likelihood under the Independence Model Criterion
Multivariate multiple imputations were performed (n=36,860). We imputed 100 datasets

- *Interaction of physical activity level with fruit and vegetable intake and smoking with alcohol consumption was assessed but statistically not significant*

Imputation model fit

In table 6, we summarized the distributional similarity of the observed and imputed observations using summary statistics and showed that the distributions of the imputed and observed values are comparable.

Table 6. Imputation model fit: comparison of summary statistics in observed and imputed datasets

Socioeconomic characteristics	Observed	Imputed dataset	P-value
**Age** (*mean/SD*)	64.9 (10.9)	64.9 (10.9)	-
**Sex** (female) (%)	55.7	55.7	-
**Living area** (%)			
Rural	36.0	35.4	0.221
Small	30.9	31.6	
Large city/town	33.1	33.0	
**Level of education** [a] (%)			
Primary	41.8	42.3	0.065
Secondary	30.6	29.6	
Higher	27.6	28.1	
**Marital status** (*with partner*) (%)	67.0	68.1	0.074
**Net income** (median/IQR)	30,463.22 (31,030.7)	31,016.36 (31,132.2)	0.061
Social support (yes) (%)	31.8	30.4	0.082
Retirement (yes) (%)	56.7	54.9	0.051
Fruit and vegetable (adequate) (%)	87.6	86.3	0.134
Vigorous PA (%)			
More than once a week	28.6	29.3	0.246
Once a week	13.0	14.2	
One to three times a month	7.5	8.4	
Hardly ever, or never	50.9	48.1	
Smoking (yes) (%)	19.9	19.0	0.046*
Regular alcohol (yes) (%)	7.8	8.1	0.027*
Hand grip strength (mean/SD)	33.6 (11.8)	33.6 (11.8)	-
BMI (mean.SD)	26.4 (4.7)	26.4 (4.7)	-
Hypertension (yes) (%)	32.2	32.2	-
High blood cholesterol (yes) (%)	27.3	27.3	-
High blood sugar (yes) (%)	10.8	10.8	-

a- Based on ISCED 1997 (level 0-2)
* *p–value<0.05*

Stratified analysis for Belgium and France

We developed a separate GEE model for the two countries to assess whether the determinants vary across countries. However, the results showed no significant variation in the effect sizes of determinants between countries and the results are presented in table 7 and 8 below.

Table 7. Comparison of the multivariable GEE models for separate dataset of Belgium and France and the total sample. The results of the whole sample is reported the main manuscript.

**Variables**	**Multivariable GEE analysis AOR [95%CI]**		
	**Total sample**	**Belgium**	**France**
**Age** (*cont.*)	1.057 [1.055–1.060]***	1.055 [1.052–1.061]***	1.058 [1.039–1.064]***
**Sex**			
Male	1	1	1
Female	0.537 [0.511–0.564]***	0.541 [0.510–0.544]***	0.571 [0.512–0.697]***
**Living area**			
Rural	1	1	1
Small	1.084 [0.995–1.137]	1.075 [0.981–1.210]	1.799 [0.812–1.204]
Large city/town	1.142 [1.069–1.182]*	1.168 [1.164–1.282]*	1.144 [1.005–1.264]*
**Level of education**			
Primary	1	1	1
Secondary	0.911 [0.860–0.966]*	0.921 [0.820–0.951]*	0.882 [0.714–1.093]
Higher	0.824 [0.790–0.900]**	0.811 [0.693–0.899]**	0.834 [0.699–0.942]*
**Marital status**			
Partner	1	1	1
Alone	1.045 [0.994–1.100]	1.033 [0.882–1.114]	1.062 [0.889–1.167]
**Annual net income**			
Lower	1	1	1
Middle	0.987 [0.945–1.031]	0.966 [0.899–1.022]	0.999 [0.788–1.115]
Upper	0.816 [0.756–0.857]**	0.815 [0.754–0.858]**	0.818 [0.639–0.906]***
**Social support**			
No	1	1	1
Yes	0.805 [0.775–0.842]***	0.787 [0.703–0.848]***	0.820 [0.788–0.896]***
**Retirement**			
No	1	1	1
Yes	1.211 [1.156–1.313]**	1.240 [1.144–1.326]**	1.199 [1.112–1.335]*

** p–value<0.05; ** p<0.01; *** p <0.001*
AOR: Adjusted odds ratio
Multivariate multiple imputations were performed

- *Interaction of age and level of education with living area, income and social support was assessed but statistically not significant.*

Table 8. Comparison the multivariable GEE models for separate dataset of Belgium and France and the total sample. The results of the whole sample is reported the main manuscript.

**Variables**	**Multivariable GEE analysis [AOR [95%CI]]**		
	**Total sample**	**Belgium**	**France**
**Age** (*cont.*)	1.045 [1.042–1.048]***	1.051 [1.039–1.069]***	1.041 [1.012–1.088]***
**Sex** *(female)*	0.418 [0.392–0.446]***	0.411 [0.370–0.468]***	0.424 [0.211–0.481]***
**Physical activity**			
No regular PA	1	1	1
Regular PA	0.687 [0.639–0.734]**	0.712 [0.644–0.780]**	0.663 [0.549–0.777]**
**Fruit and vegetable intake**			
Not adequate	1	1	1
Adequate	0.932 [0.873–0.996]*	0.912 [0.861–0.969]*	0.941 [0.866–0.998]*
**Smoking**			
No	1	1	1
Yes	1.190 [1.128–1.252]*	1.211 [1.120–1.302]*	1.175 [1.118–1.246]*
**Regular alcohol**			
No	1	1	1
Yes	1.042 [0.960–1.131]	1.029 [0.886–1.172]	1.057 [0.958–1.156]
**BMI**			
Normal	1	1	1
Overweight	1.082 [1.029–1.137]*	1.069 [0.998–1.146]	1.075 [0.999–1.149]
Obesity	1.495 [1.436–1.554]***	1.512 [1.433–1.591]***	1.479 [1.436–1.528]***
**Chronic comorbidities**			
No comorbidity	1	1	1
One comorbidity	2.231 [2.100–2.373]***	2.044 [1.994–2.194]***	2.110 [1.877–2.346]***
>=2 comorbidities	4.541 [4.269–4.831]***	4.122 [3.868–4.476]***	4.611 [4.300–4.944]***
**Grip strength** (*cont.*)	0.987 [0.984–0.990]***	0.988 [0.982–0.994]***	0.985 [0.980–0.991]***
**Depression**			
No			
Yes	1.269 [1.212–1.328]**	1.272 [1.188–1.357]**	1.260 [1.164–1.356]**

** p–value<0.05; ** p<0.01; *** p <0.001*
AOR: Adjusted odds ratio;
Multivariate multiple imputations were performed

- *Interaction of physical activity level with fruit and vegetable intake and smoking with alcohol consumption was assessed but statistically not significant*

References

Rubin, D. B. (2009). Multiple Imputation for Nonresponse in Surveys, John Wiley & Sons.

Van Buuren, S. (2018). Flexible imputation of missing data, CRC press.
