# Supplementary material for: Socioeconomic and behavioral determinants of cardiovascular diseases among older adults in Belgium and France: A longitudinal analysis from the SHARE study
Source: PLoS One. 2020 Dec 4;15(12):e0243422. doi: 10.1371/journal.pone.0243422 (PMC7717541; doi:10.1371/journal.pone.0243422)
Supplement: Attachment — Submitted filename: PONE-D-20-21259_R1 LNB.pdf [file pone.0243422.s003.pdf]

PLOS ONE

Socioeconomic and behavioral determinants of cardiovascular diseases among older adults in Belgium and France: a longitudinal analysis from the SHARE study

--Manuscript Draft--

**Manuscript Number:**	PONE-D-20-21259R1
**Article Type:**	Research Article
**Full Title:**	Socioeconomic and behavioral determinants of cardiovascular diseases among older adults in Belgium and France: a longitudinal analysis from the SHARE study
**Short Title:**	Determinants of cardiovascular diseases among older adults
**Corresponding Author:**	Hamid Yimam Hassen, MPH, MSc<br>University of Antwerp<br>Wilrijk, Antwerp BELGIUM
**Keywords:**	socioeconomic; behavioral; determinants; cardiovascular diseases; older adults
**Abstract:**	Despite advances in the healthcare system, cardiovascular diseases (CVDs) are still an important public health problem with disparities in the burden within and between countries. Studies among the adult population documented that socioeconomic and environmental factors play a role in the incidence and progression of CVDs. However, evidence is scarce on the socioeconomic determinants and the interplay with behavioral risks among older adults. Therefore, we identified socioeconomic and behavioral determinants of CVDs among older adults. Our sample consisted of 14,322 people aged 50 years and above from Belgium and France who responded to the waves 4, 5, 6 and/or 7 of the Survey of Health Ageing and Retirement in Europe. The effect of determinants on the occurrence of CVD was examined using a Generalized Estimating Equation (GEE) approach for binary longitudinal data. The overall rate of heart attack was 8.3%, which is 7.6% in Belgium and 9.1% in France. Whereas, 2.6% and 2.3% in Belgium and France, respectively, had experienced stroke. In the multivariable GEE model, older age [AOR: 1.057, 95%CI: 1.055–1.060], living in large cities [AOR: 1.14, 95%CI: 1.07–1.18], and retirement [AOR: 1.21, 95%CI: 1.16–1.31] were associated with higher risk of CVD. Furthermore, higher level of education [AOR: 0.82, 95%CI: 0.79–0.90], upper wealth quantile [AOR: 0.82, 95%CI: 0.76–0.86] and having social support [AOR: 0.81, 95%CI: 0.77–0.84] significantly lowers the odds of having CVD. A higher hand grip strength was also significantly associated with lower risk of CVD [AOR: 0.987, 95%CI: 0.984–0.990]. This study demonstrated that older adults who do not have social support, live in big cities, belong to the lowest wealth quantile, and have a low level of education have a higher likelihood of CVD. Therefore, community-based interventions aimed at reducing cardiovascular risks need to give more emphasis to high-risk retired older adults with lower education, no social support and those who live in large cities.
**Order of Authors:**	Hamid Yimam Hassen, MPH
	Hilde Bastiaens
	Kathleen Van Royen
	Steven Abrams
**Opposed Reviewers:**	
**Response to Reviewers:**	Response to Reviewers<br><br>PONE-D-20-21259<br>Socioeconomic and behavioral determinants of cardiovascular diseases among older adults in Belgium and France: a longitudinal analysis from the SHARE study<br>PLOS ONE<br><br>First of all, I wish to thank the editor and the reviewers, also on behalf of all authors, for their valuable and constructive comments to improve our manuscript. We revised the manuscript based on the comments and issues raised by the editor and the reviewers. We are confident that we have addressed all these comments adequately and we

Powered by Editorial Manager® and ProduXion Manager® from Aries Systems Corporation

therefore hope that the manuscript is accepted for publication. Find hereunder a point by point reply to the comments and questions raised by the editor and the two reviewers.

Editor
1.Please ensure that your manuscript meets PLOS ONE's style requirements, including those for file naming.

Response: Thank you for your suggestions. We have reviewed the manuscript to meet the journal requirements and we hope that the manuscript in its current form does so.

2.Please provide additional details regarding participant consent. In the ethics statement in the Methods and online submission information, please ensure that you have specified (1) whether consent was informed and (2) what type you obtained (for instance, written or verbal, and if verbal, how it was documented and witnessed). If your study included minors, state whether you obtained consent from parents or guardians. If the need for consent was waived by the ethics committee, please include this information.

Response: Thank you for your suggestions. We used publicly available data (available via the website http://www.share-project.org/data-documentation/data-documentation-tool.html) for a secondary data analysis. In order to clarify this, we have added the following sentences in line 208 - 214 of the revised version of the manuscript:
"We used publicly available data (available via the website http://www.share-project.org/data-documentation/data-documentation-tool.html) for a secondary data analysis. SHARE underwent a review of ethical standards by the University of Mannheim's internal review board (IRB). Ethical considerations including written informed consent has been taken care of by another institution. Details on the conduct of the study including the ethical approval can be found elsewhere (Alcser, Benson et al. 2005)."

Reviewer # 1
1.The work is very well conducted, the ideas are original and relevant. The results are very interesting and will be very beneficial for the scientific community. Additionally, I find it well written and easy to understand. I would just like to comment that in table 1 in the Sex (Female) variable, I noticed that there is a typo: you need to close the parentheses.

Response: Thank you for your feedback and appreciation for our work. We corrected the typo accordingly.

Reviewer # 2
1.The authors conducted a large cohort study evaluating socioeconomic determinants and cardiovascular disease using data collected from several waves of the SHARE study. By using marginal GEE models the investigators were able to account for between- and within- subject effects of social determinants. The paper was well thought out in many ways but there are some areas for improvement. Suggested edits/comments could be found below.

Response: Thank you for your feedback. The comments were very helpful and we revised the manuscript thoroughly. We hope that we addressed all your concerns in the revised manuscript.

2.Please provide some analysis of individuals for which the outcomes of interest were missing. The authors state in line 108-109 that only persons with outcome data were contained in the sample but it is not clear how not including these persons may have biased the results. Even some descriptive analyses among persons with missing outcome data would be helpful. This is exceptionally important for the outcome which may be missing a large proportion of individuals who are deceased and therefore were not included in the sample.

Response: Thank you for your feedback. As we mentioned in line 108 to 110, we

Powered by Editorial Manager® and ProduXion Manager® from Aries Systems Corporation

excluded individuals who are aged < 50 years, individuals that already have CVD at enrollment and those that did not have information on the CVD outcome variables at enrollment. Participants with intermediate missingness in terms of the outcome variables are included in the analysis and managed using the MI technique. The main reason to exclude individuals with no CVD outcome information at enrollment is to make sure that the individuals that are included are free from CVD events prior to study initiation. In total, 467 participants were excluded (108 being aged below 50 years, 117 with missing outcome values at enrollment and 242 individuals that already had a history of CVD at enrollment). Although we assume missingness of CVD outcomes at enrollment to be completely at random (MCAR) by not including those 117 participants, the percentage of missing outcome data is below 0.8% and we therefore believe that the impact on the inference is limited when the underlying missingness mechanism is mis-specified. Nevertheless, we do agree with the reviewer that a descriptive analysis to deal with the baseline missingness could be explored. As recommended by the reviewer, we performed a descriptive analysis to compare the included individuals and the individuals that were excluded due to missing outcome values (n=117) in terms of a set of covariates. The descriptive results thereof are presented in table 1 of the supplementary material. The results show that individuals who were included are comparable with the excluded ones in terms of socioeconomic characteristics. We also added a description in line 166 to 171 of the revised manuscript.

3.Related to this point, proportions of missingness in tables 3-4 were helpful, however, in general it would be helpful to know more about the multiple imputations models. How were variables selected and were there auxiliary variables used to specify the missing model?

Response: Thank you for this comment. Indeed, we used auxiliary variables in combination with the covariate information used in the substantive model(s) in order to specify the imputation models. Examples of such auxiliary variables include family size, activities of daily living, limitation of activities, self-perceived health, among other variables. The perspective taken in the Multiple Imputation by Chained Equations (MICE) approach, also referred to as full conditional specification, is one of a specification of several imputation models which sequentially imputed missing values for a given variable when regressed against all other covariates available. For more details regarding this approach and the justification of full conditional specification, we refer the reader to (Van Buuren 2018). We revised the description in line 194 to 200 of the analysis section in order to clarify this. Moreover, more details concerning the imputation models and the covariates included therein are included in page 6 of the supplementary material. In general, the inclusion of auxiliary variables in the imputation models, next to the covariates present in the substantive model(s), albeit potentially unimportant from an explanatory perspective, leads to a more precision in terms of estimation of the model parameters in the final substantive model.

4.Please clarify if any of the "multivariate". This appears to be used interchangeably but it seems as though "multivariable" may be the correct wording. The different types of models have very different implications.

Response: We agree with the reviewer that a multivariate analysis is different from a multiple or multivariable one, the latter being the analysis that has been conducted in our manuscript. Therefore, we revised the manuscript to consistently use ‘multivariable’ throughout the manuscript.

5.While the authors allude to some of the limitations of using self-reported data, it might be helpful to include some more discussion of how measurement error may have biased some of these findings (e.g., low SES underreporting certain social behaviors such as alcohol/tobacco use/abuse, how does self-reporting CV events limit this analysis?).

Response: We kindly agree with the reviewer that those in low socioeconomic status usually have a higher likelihood of under-reporting CVD status, risks and other health related issues, which might nullify the effect size of SES on CVD. This indicates the effect of SES on CVD could be even more than what we found. We elaborated more extensively on the limitation of the self-reported data and discussed this in view of the reviewer’s comment in line 367 to 374 of the revised manuscript.

Powered by Editorial Manager® and ProduXion Manager® from Aries Systems Corporation

6.The mediation analysis was well thought out but since this contains so many similar social constructs, the authors may benefit from removing it from the main text and supplement. It can be explored in future work when developing a causal model rather than the present predictive one. Collinearity also may be an issue between some of the crudely defined socioeconomic factors.

Response: We agree that the mediation analysis needs more exploration. We accept that this study and more importantly its design did not fully comply with nor allow for a formal and in-depth mediation analysis as we have a number of related determinants and mediators. Separate mediation analyses are needed, principally for each determinant-mediator-outcome relationship. We will consider to perform an extensive mediation analysis in the continuing analysis. Initially, we believed that this mediation analysis would provide some indications for future studies. However, to avoid confusion and mis-understanding for readers, we removed it from the main text and the supplement.

7.A similar analysis looking at incident CVD cases would be interesting either for this study or in the future. Is there a way to exclude persons with prevalent CVD events from earlier waves and at baseline of this study to understand if one of these factors (i.e., retirement status, depression) cause CVD in this population?

Response: Thank you for your feedback. In fact, we excluded those individuals with CVD during the first wave of their enrollment as we mentioned in line 108 to 110 of the methods section. However, in this study, as the survey is every two years, we could not ascertain the exact number of CVD events (MI, stroke, etc..) and the exact date of such an event in between the waves. This made the incidence analysis somewhat complicated. Nevertheless, we do believe that this is very interesting and a potential avenue for further research. As suggested, we will consider to perform incidence analysis in the future.

8.Reporting variability within and between countries is an advantage of using hierarchical models such as the one employed in this study but there is no mention of it in the text. Only descriptive results were presented between the countries. Please include more information on how these countries differ.

Response: Thank you for your comment. We explored the difference between two countries extensively in the revised version. Besides the descriptive statistics, we also compared the difference between countries in several ways. Firstly, we compared both countries using differences in socioeconomic as well as the outcome variable. Secondly, as we have mentioned in line 187 to 190, we developed a separate GEE model for the two countries to assess whether the determinants vary across countries. However, the results showed no significant difference in the effect sizes of determinants between countries (detailed results are indicated in table 7 and 8 of the supplementary material). Thirdly, we performed a combined analysis without the inclusion of country-specific determinants under the assumption that such differences were unimportant based on our earlier stratified analysis. Hence, we finally preferred to present the results of the (combined) GEE model, for both countries combined.

9.It will be generally important to state whether this is a predictive model that identifies risk factors and not a causal model which may be limited to confounding and selection biases.

Response: Thank you for your feedback. We do agree that the aim of this study is to identify risk factors for the occurrence of CVD, i.e. a so-called risk factor identification study. We stated this explicitly in the discussion section and indicated that our model is not a causal one, rather one that focuses on association and not on (the establishment of) causality. We believe that this study would provide overall insight for further individual causal study.

References

Alcser, K. H., G. Benson, A. Börsch-Supan, A. Brugiavini, D. Christelis, E. Croda, M. Das, G. de Luca, J. Harkness and P. Hesselius (2005). "The survey of health, aging,

	and retirement in Europe—Methodology." Mannheim Mannheim Research Institute for the Economics of Aging (MEA).<br><br>Van Buuren, S. (2018). Flexible imputation of missing data, CRC press.

Hamid Y. Hassen
Hamid.hassen@uantwerpen.be

Additional Information:	
**Question**	**Response**
**Financial Disclosure**<br><br>Enter a financial disclosure statement that describes the sources of funding for the work included in this submission. Review the submission guidelines for detailed requirements. View published research articles from *PLOS ONE* for specific examples.<br><br>This statement is required for submission and **will appear in the published article** if the submission is accepted. Please make sure it is accurate.<br><br>**Unfunded studies**<br>Enter: *The author(s) received no specific funding for this work.*<br><br>**Funded studies**<br>Enter a statement with the following details:<br>• Initials of the authors who received each award<br>• Grant numbers awarded to each author<br>• The full name of each funder<br>• URL of each funder website<br>• Did the sponsors or funders play any role in the study design, data collection and analysis, decision to publish, or preparation of the manuscript?<br>• **NO** - Include this sentence at the end of your statement: *The funders had no role in study design, data collection and analysis, decision to publish, or preparation of the manuscript.*<br>• **YES** - Specify the role(s) played.<br><br>* typeset	This study utilized data from the SHARE wave 4 to 7. The SHARE data collection is primarily funded by the European Commission (Horizon 2020), the US National Institute on Aging, and various national sources (For a full list of funding institutions, see http://www.share-project.org). This specific analysis is supported by EU funded SPICES project (Grant agreement ID: 733356). All funders had no role in the study design, data collection and analysis, decision to publish, or preparation of the manuscript.
**Competing Interests**<br><br>Use the instructions below to enter a competing interest statement for this	The authors have declared that no competing interests exist.

submission. On behalf of all authors, disclose any competing interests that could be perceived to bias this work—acknowledging all financial support and any other relevant financial or non-financial competing interests.<br><br>This statement **will appear in the published article** if the submission is accepted. Please make sure it is accurate. View published research articles from *PLOS ONE* for specific examples.<br><br>**NO authors have competing interests**<br><br>Enter: *The authors have declared that no competing interests exist.*<br><br>**Authors with competing interests**<br><br>Enter competing interest details beginning with this statement:<br><br>*I have read the journal's policy and the authors of this manuscript have the following competing interests: [insert competing interests here]*<br><br>* typeset	
**Ethics Statement**<br><br>Enter an ethics statement for this submission. This statement is required if the study involved:<br><br>• Human participants<br>• Human specimens or tissue<br>• Vertebrate animals or cephalopods<br>• Vertebrate embryos or tissues<br>• Field research<br><br>Write "N/A" if the submission does not require an ethics statement.<br><br>General guidance is provided below. Consult the submission guidelines for detailed instructions. **Make sure that all information entered here is included in the Methods section of the manuscript.**	We used publicly available data (available via the website http://www.share-project.org/data-documentation/data-documentation-tool.html) for a secondary data analysis. SHARE underwent a review of ethical standards by the University of Mannheim's internal review board. Ethical considerations including the written informed consent has been taken care of by another institution. Details on the conduct of the study including the ethical approval can be found elsewhere [28].

**Format for specific study types**<br><br>**Human Subject Research (involving human participants and/or tissue)**<br>• Give the name of the institutional review board or ethics committee that approved the study<br>• Include the approval number and/or a statement indicating approval of this research<br>• Indicate the form of consent obtained (written/oral) or the reason that consent was not obtained (e.g. the data were analyzed anonymously)<br><br>**Animal Research (involving vertebrate animals, embryos or tissues)**<br>• Provide the name of the Institutional Animal Care and Use Committee (IACUC) or other relevant ethics board that reviewed the study protocol, and indicate whether they approved this research or granted a formal waiver of ethical approval<br>• Include an approval number if one was obtained<br>• If the study involved *non-human primates*, add *additional details* about animal welfare and steps taken to ameliorate suffering<br>• If anesthesia, euthanasia, or any kind of animal sacrifice is part of the study, include briefly which substances and/or methods were applied<br><br>**Field Research**<br><br>Include the following details if this study involves the collection of plant, animal, or other materials from a natural setting:<br>• Field permit number<br>• Name of the institution or relevant body that granted permission	
**Data Availability**<br><br>Authors are required to make all data underlying the findings described fully available, without restriction, and from the time of publication. PLOS allows rare exceptions to address legal and ethical concerns. See the PLOS Data Policy and FAQ for detailed information.	Yes - all data are fully available without restriction

Powered by Editorial Manager® and ProduXion Manager® from Aries Systems Corporation

A Data Availability Statement describing where the data can be found is required at submission. Your answers to this question constitute the Data Availability Statement and **will be published in the article**, if accepted.<br><br>**Important:** Stating 'data available on request from the author' is not sufficient. If your data are only available upon request, select 'No' for the first question and explain your exceptional situation in the text box.<br><br>Do the authors confirm that all data underlying the findings described in their manuscript are fully available without restriction?	
**Describe where the data may be found in full sentences. If you are copying our sample text, replace any instances of XXX with the appropriate details.**<br><br>• If the data are **held or will be held in a public repository**, include URLs, accession numbers or DOIs. If this information will only be available after acceptance, indicate this by ticking the box below. For example: *All XXX files are available from the XXX database (accession number(s) XXX, XXX.*).<br>• If the data are all contained **within the manuscript and/or Supporting Information files**, enter the following: *All relevant data are within the manuscript and its Supporting Information files.*<br>• If neither of these applies but you are able to provide **details of access elsewhere**, with or without limitations, please do so. For example:<br><br>*Data cannot be shared publicly because of [XXX]. Data are available from the XXX Institutional Data Access / Ethics Committee (contact via XXX) for researchers who meet the criteria for access to confidential data.*<br><br>*The data underlying the results presented in the study are available*	The data underlying the results presented in the study are available from the SHARE Research Data Center (https://releases.sharedataportal.eu/releases)

from (include the name of the third party and contact information or URL).<br>• This text is appropriate if the data are owned by a third party and authors do not have permission to share the data.<br><br>* typeset	
Additional data availability information:	

Cover letter

July 7, 2020
Editorial department of PLOS ONE

Subject: Submission of Manuscript

Dear *Editor-in-Chief*;

As per your call for paper on "***Primary and Secondary Prevention of Cardiovascular Diseases***", I am submitting a manuscript entitled "**Socioeconomic and behavioral determinants of cardiovascular diseases among older adults in Belgium and France: A longitudinal analysis from the SHARE study**" for consideration of publication in PLOS ONE.

Despite studies documented risk factors of CVDs on the adult population, the evidence is scarce on the socioeconomic determinants and the interplay with behavioral risks among older adults. We felt assessing the role of socioeconomic determinants in the occurrence and progression of CVD and the interplay with behavioral and clinical factors could help to inform targeted community-based interventions. Hence, in this study, we identified the socioeconomic determinants and behavioral risks of CVDs among older adults in Belgium and France. We believe that our findings will have a substantial impact in guiding interventions aimed at decreasing the burden of CVDs among older adults in Europe.

Our results will be of great interest to the readers of the PLOS ONE, particularly those involved in prevention of CVDs. Both researchers and public health practitioners will be benefited from our findings.

I, on behalf of all authors, confirm that this work is original and has not been published, nor is it currently under consideration for publication elsewhere.
Thank you for consideration of this manuscript. We look forward to hearing from you.

Yours Sincerely,

Hamid Y. Hassen
Phone: +32466298748
Email: Hamid.Hassen@uantwerpen.be

Manuscript Click here to access/download;Manuscript;Manuscript.docx

Socioeconomic and behavioral determinants of cardiovascular diseases among older adults in Belgium and France: a longitudinal analysis from the SHARE study

Hamid Yimam Hassen[1]*, Hilde Bastiaens[1,2], Kathleen Van Royen[1,4], Steven Abrams[2,3]

[1]Department of Primary and Interdisciplinary Care, Faculty of Medicine and Health Sciences, University of Antwerp, Antwerp, Belgium

[2]Global Health Institute, Faculty of Medicine and Health Sciences, University of Antwerp, Antwerp, Belgium

[3]Interuniversity Institute for Biostatistics and statistical Bioinformatics, Data Science Institute, Hasselt University, Diepenbeek, Belgium

[4]Department of Communication Studies, Faculty of Social Sciences, University of Antwerp, Antwerp, Belgium

*Corresponding author

Email: Hamid.Hassen@uantwerpen.be (HYH)

Abstract

Despite advances in the healthcare system, cardiovascular diseases (CVDs) are still an important public health problem with disparities in the burden within and between countries. Studies among the adult population documented that socioeconomic and environmental factors play a role in the incidence and progression of CVDs. However, evidence is scarce on the socioeconomic determinants and the interplay with behavioral risks among older adults. Therefore, we identified socioeconomic and behavioral determinants of CVDs among older adults. Our sample consisted of 14,322 people aged 50 years and above from Belgium and France who responded to the waves 4, 5, 6 and/or 7 of the Survey of Health Ageing and Retirement in Europe. The effect of determinants on the occurrence of CVD was examined using a Generalized Estimating Equation (GEE) approach for binary longitudinal data. The overall rate of heart attack was 8.3%, which is 7.6% in Belgium and 9.1% in France. Whereas, 2.6% and 2.3% in Belgium and France, respectively, had experienced stroke. In the multivariable GEE model, older age [AOR: 1.057, 95%CI: 1.055–1.060], living in large cities [AOR: 1.14, 95%CI: 1.07–1.18], and retirement [AOR: 1.21, 95%CI: 1.16–1.31] were associated with higher risk of CVD. Furthermore, higher level of education [AOR: 0.82, 95%CI: 0.79–0.90], upper wealth quantile [AOR: 0.82, 95%CI: 0.76–0.86] and having social support [AOR: 0.81, 95%CI: 0.77–0.84] significantly lowers the odds of having CVD. A higher hand grip strength was also significantly associated with lower risk of CVD [AOR: 0.987, 95%CI: 0.984–0.990]. This study demonstrated that older adults who do not have social support, live in big cities, belong to the lowest wealth quantile, and have a low level of education have a higher likelihood of CVD. Therefore, community-based interventions aimed at reducing cardiovascular risks need to give more emphasis to high-risk retired older adults with lower education, no social support and those who live in large cities.

Introduction

Cardiovascular diseases (CVDs) are the leading cause of morbidity and mortality worldwide and constitute a major burden on the healthcare system in all countries [1]. In 2017, an estimated 422.7 million prevalent cases, 17.9 million deaths, and 366 million disability adjusted life years (DALYs) were attributed to CVDs [2]. In Europe, nearly half of all deaths are related to CVDs so being responsible for more deaths than any other condition [3, 4]. In Belgium and France, CVDs account for 28% and 24% of all deaths respectively [5].

Despite advances in the primary and secondary prevention, there are still disparities in the CVD burden within and across countries, some segment of the population being at a higher risk [6-8]. Previously, epidemiological studies have focused on identifying and modifying individual risk factors. As a result, several health conditions and behavioral factors such as tobacco use, unhealthy diet, alcohol consumption and physical inactivity were identified to be related with CVDs. The reduction of such unhealthy lifestyles and early detection and treatment of risk factors are effective to reduce the burden. Hence, improvements have been observed in the reduction of CVD morbidities and its associated premature mortality among adults. However, many cardiovascular risks still remain as a major public health issue at different rates globally. The burden of CVDs highly varied across segments of the population, older age, the least affluent and deprived communities are disproportionately affected [9-11]. Socioeconomic and environmental factors, including income, living condition, level of education also play an important role in the development, progression and outcomes of CVDs [12-15].

As part of the efforts to narrow disparities, the World Health Organization (WHO) established a Commission on Social Determinants of Health and developed a conceptual framework for action [16]. The framework emphasizes that social determinants as well as resources to prevent illness, are not distributed randomly throughout human society and such disparities need to be

identified. Albeit some studies assessed the socioeconomic determinants of CVDs in the general population, the evidence specific to older adults is scant. Older adults have a different lifestyle and social dynamics compared to younger counterparts [17]. As age increases, the shrinkage of social networks through deaths of friends puts older people at risk of social isolation and loneliness, which might be associated with the occurrence of CVDs in those individuals [18].

Identifying the socioeconomic and behavioral factors of CVDs and evaluating the moderation effect in between could help in improving intervention strategies to reduce the burden of CVD in the older population. The EU Horizon 2020 funded project named SPICES – Scaling-up Packages of Interventions for cardiovascular diseases in Europe and Sub-Saharan Africa, aims to reduce CVD risks among vulnerable populations through risk profiling and coaching intermediate-risk participants. The current study supports the identification of target population and area of intervention among high-risk older adults in Belgium and France. Hence, in this study, we identified the socioeconomic determinants and behavioral risks of CVDs among older adults in Belgium and France, using a longitudinal data collected in the Survey of Health, Ageing and Retirement in Europe (SHARE) study. We also assessed the moderation and interplay of socioeconomic, behavioral and clinical risk factors on CVD, particularly heart attack and stroke.

Methods and materials

Study design and participants

We used the longitudinal dataset from the SHARE survey, which collects information on health status, behavioral risks, socioeconomic condition, and social networks of individuals aged 50 years and above and their partners. Details about the sampling design, methodology, and questionnaires of the SHARE survey have been presented elsewhere [19]. Methods specific to

this study are briefly summarized here. A population-based sample of the non-institutionalized population is used for this survey, with data collected via multi-stage sampling. We used data from wave 4 (2011–12), wave 5 (2013), wave 6 (2015) and wave 7 (2017) for both Belgium and France. Data from wave 3 (2008) were not included, as these data assessed the life histories of participants and did not provide the necessary information regarding health and behavioral risks. Moreover, due to the wide time interval between waves 1 (2004) and 2 (2006) on the one hand and wave 4 (2011) and subsequent waves on the other hand (five years), thereby resulting in interruption of the longitudinal nature of the data, we relied on data derived from waves 4 to 7.

Participants fulfilling the following criteria were included to the analysis: i) aged 50 years or above and ii) having information on the main outcome variables, heart attack, stroke or atherosclerosis at least in one wave. Data were not analyzed for participants i) < 50 years of age and ii) people who were reported as having CVD during enrollment, and iii) with no CVD information at enrollment. Overall, 7,914 adults in Belgium and 6,408 in France participated in at least one wave of the survey and were eligible, resulting in a total number of person-observations of 20,555 and 16,305 for Belgium and France, respectively.

Outcome and exposure assessment

Cardiovascular diseases

The presence or absence of cardiovascular diseases was measured in the SHARE study using the question '*Has a doctor told you that you had/Do you currently have any of the conditions on this card?*'. The possible answers were: yes or no; if yes, the time of first diagnosis was asked. In this study, we defined a person as having CVD if he/she has at least one of the following problems, or both; i) a heart attack including myocardial infarction or coronary thrombosis; or ii) a stroke or cerebrovascular disease diagnosed between waves.

Socioeconomic determinants

Socioeconomic variables, including age, sex, household income, retirement status, marital status, educational status, and social support were collected in the SHARE study. In our study, we categorized marital status as a dichotomous variable as living with a partner or living alone, where the latter included those who were widowed, divorced or never married. Level of education was assessed with the International Standard Classification of Education (ISCED) 1997 [20], and was categorized as low (level 0-2), medium (levels 3 and 4), and high (levels 5 and 6). We categorized wealth using three quantiles for each country into upper, middle and lower one-third. We categorized living areas in three groups: i) large town or city, ii) small town, and iii) rural or village. Social support was measured using two questions: (1) '*In the past twelve months, has any family member from outside the household, friend, or neighbor given you any type of support?*' and, if 'yes', (2) '*Is there someone living in this household who has helped you regularly during the past twelve months*?'. We dichotomized this information by defining social support as having positive responses on both of the above questions, and no social support otherwise.

Behavioral determinants

Smoking status in the SHARE survey was assessed by asking '*Do you smoke at the present time?'*. Regular alcohol consumption was assessed with the question '*In the last three months, how often did you have six or more units of alcoholic beverages on one occasion?*'. The possible response options were: i) daily or almost daily, ii) 5 or 6 days a week, iii) 3 or 4 days a week, iv) once or twice a week, v) once or twice a month, vi) less than once a month and vii) not at all in the last 3 months. We dichotomized this variable into regular alcohol consumption (i-iv) and no regular alcohol consumption (v-vii). The frequency of engaging in vigorous and moderate physical activity (PA) was assessed asking: '*How often do you engage in vigorous/moderate PA, such as sports, heavy housework, or a job that involves physical*

labor?' The possible responses were: i) more than once a week, ii) once a week, iii) once to three times a month, and iv) hardly ever or never. We dichotomized this variable into regular PA (i and ii), and no regular PA (iii and iv). Fruit and vegetable intake was assessed by asking: '*In a regular week, how often do you have a serving of fruit and vegetables?'*. The possible responses were: i) every day, ii) three to six times a week, iii) twice a week, iv) once a week, v) less than once a week. We dichotomized into adequate (i) and inadequate intake (ii-v).

Physical measurements and clinical characteristics

We computed body mass index (BMI) using the self-reported values of body weight and height in each wave. Hand-grip strength was measured by trained interviewers using a handheld dynamometer. Self-reported long-term illnesses were assessed with the question '*Do you have any long-term health problems, illness, disability or infirmity such as chronic respiratory, kidney disease, cancer, etc.?*'. We categorized comorbidities into; i) no comorbidity, ii) one comorbidity, and iii) two and more comorbidities. Depression status was assessed using a EURO-12, a 12-item geriatric depression scale and those who scored below 4 were considered as not having depression and those above 4 as having depression. Moreover, whether the participant ever had CVD risks, including high blood pressure or hypertension; high blood cholesterol; and diabetes and/or high blood sugar were assessed.

Statistical analysis

Descriptive statistics including absolute and relative frequencies for categorical variables, mean with Standard Deviation (SD) or median with Inter Quartile Range (IQR) for continuous variables were employed to summarize the characteristics of participants. We compared socioeconomic characteristics between individuals included in the analysis (n = 14,322) and those excluded as a result of missing CVD outcome values at enrollment (n = 117), using two sample t-tests and chi-square tests for numerical and categorical variables, respectively. The

results show that individuals who were included are comparable with the excluded ones in terms of socioeconomic characteristics (*table 1 in S1 Appendix*). To estimate the population averaged effect of socioeconomic, behavioral and physical determinants on the occurrence of CVD, we developed two independent marginal models using multivariable Generalized Estimating Equations (GEEs) with the binomial distribution and logit link function [21]. GEE accounts for within-subject association and allows to estimate the between-subject effects of the relationships of determinants with the occurrence of CVD over the observation period. The unequal selection probabilities and unequal numbers of follow-up interviews among study participants was taken into consideration in the GEE analysis using individual survey weights. We assumed that the within-subject association among the vectors of repeated outcomes would have an exchangeable working correlation structure. Albeit other correlation structures were considered, goodness-of-fit indices were found under the exchangeable correlation structure (*table 3 in S1 Appendix*). As a goodness-of-fit indices, we used the quasi-likelihood under the Independence Model Criterion (QIC), the Wald $\chi 2$ test, and we performed a residual analysis to assess the presence of outliers and their random distribution [22, 23]. To compare the models, we used both the QIC and the Wald-test between the null, non-adjusted model and the adjusted models. Hence, the model with an exchangeable correlation structure was selected as it provided the smallest QIC. To evaluate whether the determinants vary between Belgium and France, we also performed a stratified analysis fitting a model for each country separately, however, there was no significant difference between countries in terms of covariate effects and the results are shown in the supplement (*table 7 and 8 in annex 1*). We performed the GEE analyses using the '**geepack**' package of the free statistical software, R version 3.6.1 [24].

The percentage of missing values across all the variables varied between 0 and 37.6%. Missing results were imputed for all missing variables evaluated in the GEE model. Assuming the missing data to be missing at random (MAR), we performed Multivariate Imputation by

Chained Equations (MICE) using the '**mice'** package in R [25]. We imputed the dataset to have M = 100 complete datasets using all the variables included in the model and some additional auxiliary variables (detailed description on the imputation model is available in the supplementary material). For more details regarding the MICE approach we used, we refer the reader to a book by Van Buuren [26]. The parameters of interest were estimated in each imputed dataset separately, and combined using Rubin's rules [27]. We performed a sensitivity analysis to compare the results obtained from a multiple imputation approach with those from a complete case analysis. The results were comparable between the two approaches, except in terms of precision of the estimated model parameters. More specifically, multiple imputation was found to lead to more precise estimates which can be seen from the shorter confidence intervals (*table 4 and 5 in S1 Appendix)*. We assessed the distributional similarity of the observed and imputed observations using summary statistics and showed that the distributions of the imputed and observed values are comparable (*table 6 in S1 Appendix*).

Ethics statement

We used publicly available data (available via the website http://www.share-project.org/data-documentation/data-documentation-tool.html) for a secondary data analysis. SHARE underwent a review of ethical standards by the University of Mannheim's internal review board. Ethical considerations including the written informed consent has been taken care of by another institution. Details on the conduct of the study including the ethical approval can be found elsewhere [28].

Results

Socioeconomic characteristics participants

Table 1 displays the socioeconomic characteristics of participants at their first enrollment. The mean age of participants was 64.4 (SD: 10.9) and 65.6 (SD: 10.9) in Belgium and France,

respectively. Overall, 55.7% were females and 41.8% had low level of education. Majorities (67.0%) were living with their partners and one-third (33.1%) were living in large towns or cities. More than half (55.4%) were retired and nearly one-third (31.8%) had regular social support from family members or any other person. The median household annual net income was 32.6 and 28.6 thousand Euro in Belgium and France, respectively.

Table 1. Socioeconomic characteristics of study participants during enrollment to interview ~~stratified~~ by country ~~(n=14,322)~~.

Participant characteristics	Total	Belgium	France
**Age** *(years)*	64.9 (10.9)	64.4 (10.9)	65.6 (10.9)
**Sex** *(female)*	7975 (55.7)	4340 (54.8)	3635 (56.7)
**Level of education** [a] (n=14,130)			
Low	5902 (41.8)	3135 (40.0)	2767 (44.0)
Medium	4327 (30.6)	2121 (27.0)	2206 (35.0)
High	3901 (27.6)	2579 (32.9)	1322 (21.0)
**Marital status** (n=14,250)			
With partner	9547 (67.0)	5338 (67.6)	4209 (66.2)
Alone	4703 (33.0)	2553 (32.4)	2150 (33.8)
**Living area** (n=13,793)			
Rural	4965 (36.0)	2088 (27.5)	2877 (46.5)
Small town	4263 (30.9)	2632 (34.6)	1631 (26.3)
Large town or city	4565 (33.1)	1631 (26.3)	1683 (27.2)
**Family size (no of persons)**			
One	3869 (27.0)	2071 (26.2)	1798 (28.1)
Two	7989 (55.8)	4326 (54.7)	3663 (57.2)
Three	1493 (10.4)	923 (11.7)	570 (8.9)
Four and above	971 (6.8)	594 (7.5)	377 (5.9)
**Estimated household income** (€) (median(IQR)) (n=14,109)	30463.2 (31030.7)	32640.3 (35174.8)	28621.0 (26953.5)
Lower	4404 (31.2)	2377 (30.6)	2027 (32.0)
Middle	4452 (31.6)	2421 (31.1)	2031 (32.1)
Upper	5253 (37.2)	2984 (38.3)	2269 (35.9)
**Social support** (n=11,275)			
Yes	3589 (31.8)	2166 (34.7)	1423 (28.3)
No	7686 (68.2)	4083 (65.3)	3603 (71.7)
**Retirement** (n=13,994)			
Yes	7939 (56.7)	3971 (51.7)	3968 (62.9)
No	6055 (43.3)	3717 (48.3)	2338 (37.1)

a- Based on ISCED 1997 (level 0-2)

Behavioral, physical measurements and other health related characteristics

The trend of behavioral, physical and other health related characteristics of participants at each wave of data collection is presented in detail in the supplementary material (*table 2 in S1 Appendix*). Overall, 87.6% of participants reported adequate fruit and vegetable intake, i.e., at least one serving per day and the trend was consistent from 2011 to 2017. Above half (50.9%) hardly ever or never involved in vigorous physical activities, whereas, 65.6% reported that they performed moderate physical activities more than once a week. Similarly, the frequency of vigorous and moderate physical activity remains relatively constant across time. Overall, only 7.8% have a history of regular alcohol consumption within 6 months prior to data collection. Overall, one-fifth (19.9%) were smokers at the time of each interview and the proportion was highest in 2017 (29.2%). The rate of obesity is relatively consistent throughout the observation period, ranging 18.6% (2011) to 20.7% (2015). The overall mean of hand grip strength was 33.6 (SD: 11.8) and it was consistent across the waves. The rate of depression ranged from 29.9% in 2017 to 31.5% in 2015, with an overall rate of 30.9%.

The prevalence of cardiovascular risks

Table 2 summarizes the prevalence of CVD risks in Belgium and France. One-third (33.2%) and 31.0% of older adults in Belgium and France, respectively, had a history of hypertension at least once in their life time. The prevalence of high blood cholesterol in Belgium (30.6%) is higher than in France (23.3%). Whereas, the prevalence of high blood sugar in Belgium (10.8%) is nearly equal to the prevalence in France (10.9%).

Table 2. The prevalence of cardiovascular risks ~~stratified~~ by country ~~(n=14276)~~

**Cardiovascular risks**	**Total n(%)**	**Belgium n(%)**	**France n(%)**
Ever had hypertension *(Yes)*	4596 (32.2)	2617 (33.2)	1979 (31.0)
Ever had high blood cholesterol *(Yes)*	3898 (27.3)	2410 (30.6)	1488 (23.3)
Ever had high blood sugar *(Yes)*	1546 (10.8)	848 (10.8)	698 (10.9)
Total	14276	7888	6388

The occurrence of cardiovascular events

The overall rate of heart attack was 8.3%, which is 7.6% in Belgium and 9.1% in France. The rate of stroke was 2.6% and 2.3% in Belgium and France, respectively. Throughout the observation period, the overall rate of CVD was 9.5% and 10.7% in Belgium and France, respectively. (Table 3)

Table 3. The occurrence of cardiovascular diseases ~~stratified~~ by country (n=36,781 person observation points; Belgium=20,516 and France=16,265).

CVD events	Overall percentage	Belgium N (%)	France N (%)
Heart attack	8.3	1563 (7.6)	1483 (9.1)
Stroke	2.4	526 (2.6)	369 (2.3)
Heart attack or stroke	10.0	1,954 (9.5)	1,738 (10.7)

Socioeconomic determinants of cardiovascular diseases among older adults

In the multivariable GEE model, the odds of having CVD was 5.7% higher for a one year increase in age [AOR: 1.057, 95%CI: 1.055–1.060]. The risk of CVD is significantly higher among those who were living in large cities [AOR: 1.14, 95%CI: 1.07–1.18] than those living in rural areas. Similarly, retired older adults showed a higher odds of CVD than those who were not retired [AOR: 1.21, 95%CI: 1.16–1.31]. Being female [AOR: 0.54, 95%CI: 0.51–0.56], high level of education [AOR: 0.82, 95%CI: 0.79–0.90], higher income [AOR: 0.82, 95%CI: 0.76–0.86] and social support [AOR: 0.81, 95%CI: 0.77–0.84] imply a significantly lower risk of CVD as compared to their respective reference categories. (Table 4)

Table 4. ~~Multivariable GEE models estimating~~ the effect of socioeconomic characteristics on CVD among older adults in Belgium and France.

Variables	Percent [illegible]h outcome	COR [95%CI]	AOR [95%CI]
**Age**	*Cont.*	1.063 [1.059–1.067]***	1.057 [1.055–1.060]***
**Sex**			
Male	12.6	~~1~~	1
Female	8.0	0.60 [0.55–0.65]***	0.54 [0.51–0.56]***
**Living area**			
Rural	9.6	1	1
Small	10.4	1.08 [0.99–1.18]	1.08 [0.99–1.14]
Large city/town	10.5	1.14 [1.01–1.22]*	1.14 [1.07–1.18]*
**Level of education**			
Primary	12.7	1	1
Secondary	8.6	0.64 [0.58–0.72]**	0.91 [0.86–0.97]*
Higher	7.8	0.58 [0.52–0.65]***	0.82 [0.79–0.90]**
**Marital status**			
Partner	9.2	1	1
Alone	11.5	1.30 [1.19–1.41]*	1.04 [0.99–1.10]
**Net income**			
Lower	12.1	1	1
Middle	10.3	0.86 [0.79–0.94]*	0.99 [0.94–1.03]
Upper	7.0	0.60 [0.55–0.67]***	0.82 [0.76–0.86]**
**Social support**			
No	12.2	1	1
Yes	7.0	0.63 [0.58–0.69]***	0.81 [0.77–0.84]***
**Retirement**			
No	5.8	1	1
Yes	13.0	2.10 [1.92–2.28]***	1.21 [1.16–1.31]**

** p–value<0.05; ** p<0.01; *** p <0.001*
AOR: Adjusted odds ratio; COR: Crude odds ratio;
Multivariate multiple imputations were performed (n=36,860)

- *Interaction of age and level of education with living area, income and social support was assessed but statistically not significant.*

Behavioral and physical determinants of cardiovascular diseases among older adults

In the multivariable GEE model (Table 5), on average the odds of CVD is 31% lower for those who do regular physical activity than those who do not [AOR: 0.69, 95%CI: 0.64–0.73]. Similarly, adequate fruit and vegetable intake is also associated with lower odds of CVD among older adults [AOR: 0.93, 95%CI: 0.87–0.99]. The odds of having CVD is 1.5 times higher for those who are obese than for those not obese [AOR: 1.49, 95%CI: 1.44–1.55]. Those who have

one comorbidity and two or more multimorbidity are 2.2 and 4.5 times respectively, higher likelihood of having CVD than those with no comorbidity. The odds of having CVD is also 1.3 times higher for those who have depression than their counterparts [AOR: 1.27, 95%CI: 1.21–1.33]. Moreover, hand grip strength is significantly associated with lower CVD [AOR: 0.987, 95%CI: 0.984–0.990].

Table 5. Multivariable GEE model estimating the effect of behavioral and physical determinants of cardiovascular diseases among older adults in Belgium and France

Variables	Percent with outcome	COR [95%CI]	AOR [95%CI]
**Age**	*Cont.*	1.063 [1.059–1.067]***	1.045 [1.042–1.048]***
**Sex** *(female)*	8.0	0.60 [0.55–0.65]***	0.42 [0.39–0.45]***
**Physical activity**			
No regular PA	12.6	1	1
Regular PA	6.1	0.55 [0.51–0.60]***	0.69 [0.64–0.73]**
**Fruit and vegetable intake**			
Not adequate	10.2	1	1
Adequate	9.3	0.93 [0.82–1.06]	0.93 [0.87–0.99]*
Smoking			
No	9.1	1	1
Yes	7.9	1.16 [1.02–1.31]*	1.19 [1.13–1.25]*
**Regular alcohol consumption**			
No	11.4	1	1
Yes	10.8	0.99 [0.80–1.21]	1.04 [0.96–1.13]
**BMI**			
Normal	8.2	1	1
Overweight	10.0	1.20 [1.10–1.31]***	1.08 [1.03–1.14]*
Obesity	13.9	1.67 [1.50–1.85]***	1.49 [1.44–1.55]***
**Chronic comorbidities**			
No comorbidity	2.1	1	1
One comorbidity	8.1	2.84 [2.52–3.21]***	2.23 [2.10–2.37]***
>=2 comorbidities	21.9	6.98 [6.20–7.87]***	4.54 [4.27–4.83]***
**Grip strength**	*cont.*	0.984 [0.980–0.988]***	0.987 [0.984–0.990]*
**Depression**			
No	8.2	1	1
Yes	13.0	1.51 [1.40–1.63]***	1.27 [1.21–1.33]**

** p–value<0.05; ** p<0.01; *** p <0.001*
AOR: Adjusted odds ratio; COR: Crude odds ratio; BMI: Body Mass Index
Multivariate multiple imputations were performed (n=36,860).

- *Interaction of physical activity level with fruit and vegetable intake and smoking with alcohol consumption was examined but statistically not significant*

Discussion

Based on a large sample of adults aged 50 years and above in Belgium and France, this study aimed to identify the socioeconomic, behavioral and physical determinants of cardiovascular diseases and the interplay in between longitudinally. Whilst several studies evidenced the role of socioeconomic factors on CVD among adults, the role of these factors on older people were not well documented. This study showed that socioeconomic characteristics, including having social support, level of education, living area, retirement and income level were independent determinants of CVD among older adults.

After adjustment for the socioeconomic and other related variables, the risk of having CVD was significantly higher among older adults who live in large cities than those who live in villages. A lower green area in urban settings could be a possible reason as indicated by Seo S. and colleagues who found adults living in areas with greater amounts of green space found to have a lower risk of CVD [29]. Consistently, various studies showed that exposure to rural green space is associated with a reduction in indicators of CVD risk factors compared to urban streets [30-34]. The higher risk of CVD in the most deprived groups is more pronounced in urban areas with low amounts of green space coverage [35, 36]. Furthermore, the higher prevalence of smoking, physical inactivity, and unhealthy diet in urban areas could also be the possible reasons for higher risk of CVD. Thus, interventions aimed at reduction of CVD risks need to integrate with other sectors for optimal intervention effect.

In our study, older adults with regular social support from family or anyone else have a lower risk of CVD. This finding is similar to a study by Rosengren AL. *et al,* which indicate low social support is associated with coronary heart diseases [37]. Several studies also documented the role of social support in CVD incidence and mortality [38-40]. Both objective social isolation and the subjective perception of being isolated have been shown to be associated with

a higher rate of CVD [41, 42]. Orth-Gomer explained the psychological mechanisms of social support leading to CVD morbidity and mortality [43]. Therefore, it is essential to elucidate the molecular mechanisms of loneliness leading to CVDs. Moreover, community based CVD prevention strategies need to consider the role of social support to prevent the detrimental effects of isolation.

The present study showed, retired older adults have a higher risk of CVD after adjusted for age, sex and other socioeconomic characteristics. A recent systematic review indicated the impact of retirement on the rate of CVDs and risk factors varies across countries, in which studies in the European countries showed a detrimental effect of retirement on CVDs [44]. Pedron and colleagues identified male and low-educated retirees as potential high-risk groups for worsening CVD risk factors after retirement [45]. Retirement has been linked to increased leisure time activities but, it may reduce transport and work related activities. The effect of retirement also varied with the type of job a person retired from. A study by Godard and associates showed retirement caused an increase in the likelihood of being obese among men retiring from strenuous jobs [46]. On the other hand Stenholm and colleagues indicated retirement was associated with slight weight loss in men retiring from sedentary jobs [47]. Hence, policies concerning the retirement age need to focus on ensuring whether they are suited to individuals and contexts.

In this study, higher income and higher level of education are associated with lower risk of CVDs. A multi-country study showed cardiovascular events were more common among those with low levels of education [48]. Dégano and her colleagues also indicated the rate of CVD events is 50% lower for those with university education compared to primary or lower education [49] The variation in CVD incidence and mortality based on the socioeconomic status has also been documented elsewhere [12, 14, 15, 50].

Various physical factors were also found significant in our study. Grip strength is associated with CVD after adjusting for socioeconomic and behavioral factors. Previous studies showed the association of hand grip strength with heart failure and cardiovascular risks such as high blood pressure, high blood sugar and lipid levels [51, 52]. Hand grip strength in patients with type 2 diabetes is inversely associated with CVD independently from well-established cardiovascular risks [53]. A prospective study from the UK also showed hand grip strength has inverse associations with incidence of cardiovascular events [54]. This implies hand grip strength could be a valuable CVD risk assessment tool for older adults in combination with the already available indicators.

Our findings also showed behavioral factors, including physical inactivity, inadequate fruit and vegetable intake, obesity, smoking and depression are associated with higher risk of cardiovascular events, as it is evidenced from several studies [55, 56], indicating older adults are not an exception in this regard.

The findings from this study need to be interpreted in the context of the following limitations. First, we used the existing measures from the SHARE study and were not able to include more measures of determinants that might be more appropriate to estimate the effect of behavioral determinants on CVD. For instance, we could not explore the amount of serving of fruit and vegetable intake, the duration and intensity of vigorous and moderate physical activity level, and the amount of alcohol consumption. Secondly, the measurement of socioeconomic characteristics, CVDs and risks was self-reported. We did not assess it using physical or laboratory measurements. Participants in low socioeconomic status (SES) could under-report their CVD status and risks [57], which might nullify the effect size of SES on CVD. This indicates the effect could even be more than what we found. Further studies with a more objective assessment of CVDs and risks might provide a larger effect size. Nevertheless, SHARE used cards with a list of disease conditions to probe participants, which could minimize

the reporting bias. Thirdly, as our aim was not to identify a single determinant, we did not perform a comprehensive mediation analysis for each determinant-mediator-outcome relationship. We recommend future studies to identify independent socioeconomic and behavioral determinants of CVDs with extensive mediation analysis.

Conclusions

This study addressed several aspects of cardiovascular disease prevention areas among older adults. Our research demonstrated that older adults who are retired, do not have social support, live in big cities, belong to the lowest wealth quantile and have a low educational level have a higher likelihood of CVD. It also showed that behavioral risks are prevalent in older adults living in Belgium and France and associated with an increase in CVD as documented from our study and several other studies. Physical factors such as a better hand grip strength is associated with lower incidence of CVD. The findings from this study underlined the fact that socioeconomic disparities affect the occurrence of CVDs in older adults. Therefore, community based interventions aimed at improving cardiovascular risks need to give more emphasis to high-risk older adults to get optimal benefit from the interventions. Older adults who live in large cities with no social support need to gain more emphasis to halt the continued problem of CVD and associated premature mortality.

References

1. Roth GA, Johnson C, Abajobir A, Abd-Allah F, Abera SF, Abyu G, et al. Global, Regional, and National Burden of Cardiovascular Diseases for 10 Causes, 1990 to 2015. J Am Coll Cardiol. 2017;70(1):1-25. Epub 2017/05/17. doi: 10.1016/j.jacc.2017.04.052. PubMed PMID: 28527533.
2. WHO. A global factsheet on cardiovascular diseases 2017 [Jan 20, 2020]. Available from: https://www.who.int/news-room/fact-sheets/detail/cardiovascular-diseases-(cvds).
3. Townsend N, Wilson L, Bhatnagar P, Wickramasinghe K, Rayner M, Nichols M. Cardiovascular disease in Europe: epidemiological update 2016. European heart journal. 2016;37(42):3232-45. Epub 2016/08/16. doi: 10.1093/eurheartj/ehw334. PubMed PMID: 27523477.
4. WHO regional office for Europe. Cardiovascular Diseases; Data and Statistics 2018 [Feb 1, 2020]. Available from: http://www.euro.who.int/en/health-topics/noncommunicable-diseases/cardiovascular-diseases/data-and-statistics.

5. EUROstat. Causes of death — diseases of the circulatory system, residents 2016. Available from: https://ec.europa.eu/eurostat/statistics-explained/index.php?title=Causes_of_death_statistics.
6. Marmot M, Wilkinson R. Social determinants of health: OUP Oxford; 2005.
7. Menotti A, Puddu PE, Lanti M, Kromhout D, Blackburn H, Nissinen A. Twenty-five-year coronary mortality trends in the seven countries study using the accelerated failure time model. Eur J Epidemiol. 2003;18(2):113-22. Epub 2003/05/08. doi: 10.1023/a:1023092415353. PubMed PMID: 12733832.
8. Chow CK, Lock K, Teo K, Subramanian SV, McKee M, Yusuf S. Environmental and societal influences acting on cardiovascular risk factors and disease at a population level: a review. Int J Epidemiol. 2009;38(6):1580-94. Epub 2009/03/06. doi: 10.1093/ije/dyn258. PubMed PMID: 19261658; PubMed Central PMCID: PMCPMC2786248.
9. North BJ, Sinclair DA. The intersection between aging and cardiovascular disease. Circ Res. 2012;110(8):1097-108. Epub 2012/04/14. doi: 10.1161/circresaha.111.246876. PubMed PMID: 22499900; PubMed Central PMCID: PMCPMC3366686.
10. Yazdanyar A, Newman AB. The burden of cardiovascular disease in the elderly: morbidity, mortality, and costs. Clin Geriatr Med. 2009;25(4):563-77, vii. Epub 2009/12/01. doi: 10.1016/j.cger.2009.07.007. PubMed PMID: 19944261; PubMed Central PMCID: PMCPMC2797320.
11. Benjamin Emelia J, Muntner P, Alonso A, Bittencourt Marcio S, Callaway Clifton W, Carson April P, et al. Heart Disease and Stroke Statistics—2019 Update: A Report From the American Heart Association. Circulation. 2019;139(10):e56-e528. doi: 10.1161/CIR.0000000000000659.
12. Havranek Edward P, Mujahid Mahasin S, Barr Donald A, Blair Irene V, Cohen Meryl S, Cruz-Flores S, et al. Social Determinants of Risk and Outcomes for Cardiovascular Disease. Circulation. 2015;132(9):873-98. doi: 10.1161/CIR.0000000000000228.
13. Psaltopoulou T, Hatzis G, Papageorgiou N, Androulakis E, Briasoulis A, Tousoulis D. Socioeconomic status and risk factors for cardiovascular disease: Impact of dietary mediators. Hellenic Journal of Cardiology. 2017;58(1):32-42. doi: https://doi.org/10.1016/j.hjc.2017.01.022.
14. Lemstra M, Rogers M, Moraros J. Income and heart disease: Neglected risk factor. Can Fam Physician. 2015;61(8):698-704. PubMed PMID: 26836056.
15. Lewis MW, Khodneva Y, Redmond N, Durant RW, Judd SE, Wilkinson LL, et al. The impact of the combination of income and education on the incidence of coronary heart disease in the prospective Reasons for Geographic and Racial Differences in Stroke (REGARDS) cohort study. BMC public health. 2015;15(1):1312. doi: 10.1186/s12889-015-2630-4.
16. Solar O, Irwin A. A conceptual framework for action on the social determinants of health. Social Determinants of Health Discussion Paper 2 (Policy and Practice). Geneva: World Health Organization, 2010.
17. Smedley BD, Syme SL. Institute of Medicine (US), Committee on Capitalizing on Social Science and Behavioral Research to Improve the Public's Health, 2000. Promoting health: intervention strategies from social and behavioral research, xiv National Academy Pr, Washington, DC.
18. Leigh-Hunt N, Bagguley D, Bash K, Turner V, Turnbull S, Valtorta N, et al. An overview of systematic reviews on the public health consequences of social isolation and loneliness. Public Health. 2017;152:157-71. doi: https://doi.org/10.1016/j.puhe.2017.07.035.
19. Survey of Health, Ageing and Retirement in Europe. 2017. Available at: http://www.share-project.org (20 February 2020, date last accessed).
20. UNESCO. International Standard Classification of Education 1997. [cited 2020 May 8]. Available from: http://www.unesco.org/education/information/nfsunesco/doc/isced_1997.htm.
21. Zeger SL, Liang K-Y, Albert PS. Models for Longitudinal Data: A Generalized Estimating Equation Approach. Biometrics. 1988;44(4):1049-60. doi: 10.2307/2531734.
22. Pan W. Akaike's Information Criterion in Generalized Estimating Equations. Biometrics. 2001;57(1):120-5. doi: 10.1111/j.0006-341X.2001.00120.x.
23. Hardin JW, Hilbe JM. Generalized Estimating Equations: Chapman & Hall/CRC. Boca Raton, Florida. 2003.
24. Halekoh U, Højsgaard S, Yan J. The R package geepack for generalized estimating equations. Journal of Statistical Software. 2006;15(2):1-11.
25. Van Buuren S, Groothuis-Oudshoorn K. mice: multivariate imputation by chained equations in RJ Stat. Softw. 45, 1–67. 2011.

26. Van Buuren S. Flexible imputation of missing data: CRC press; 2018.
27. Rubin DB. Multiple Imputation for Nonresponse in Surveys: John Wiley & Sons; 2009.
28. Alcser KH, Benson G, Börsch-Supan A, Brugiavini A, Christelis D, Croda E, et al. The survey of health, aging, and retirement in Europe—Methodology. Mannheim Mannheim Research Institute for the Economics of Aging (MEA). 2005.
29. Seo S, Choi S, Kim K, Kim SM, Park SM. Association between urban green space and the risk of cardiovascular disease: A longitudinal study in seven Korean metropolitan areas. Environment International. 2019;125:51-7. doi: https://doi.org/10.1016/j.envint.2019.01.038.
30. Lanki T, Siponen T, Ojala A, Korpela K, Pennanen A, Tiittanen P, et al. Acute effects of visits to urban green environments on cardiovascular physiology in women: A field experiment. Environmental research. 2017;159:176-85.
31. Grazuleviciene R, Vencloviene J, Kubilius R, Grizas V, Dedele A, Grazulevicius T, et al. The effect of park and urban environments on coronary artery disease patients: a randomized trial. BioMed research international. 2015;2015.
32. Kardan O, Gozdyra P, Misic B, Moola F, Palmer LJ, Paus T, et al. Neighborhood greenspace and health in a large urban center. Scientific reports. 2015;5:11610.
33. Nowicki GJ, Ślusarska B, Piasecka H, Bartoszek A, Kocka K, Deluga A. The Status of Cardiovascular Health in Rural and Urban Areas of Janów Lubelski District in Eastern Poland: A Population-Based Study. Int J Environ Res Public Health. 2018;15(11):2388. doi: 10.3390/ijerph15112388. PubMed PMID: 30373289.
34. Sarrafzadegan N, Talaei M, Kelishadi R, Toghianifar N, Sadeghi M, Oveisgharan S, et al. The influence of gender and place of residence on cardiovascular diseases and their risk factors. The Isfahan cohort study. Saudi Med J. 2012;33(5):533-40. Epub 2012/05/17. PubMed PMID: 22588815.
35. Mitchell R, Popham F. Effect of exposure to natural environment on health inequalities: an observational population study. The lancet. 2008;372(9650):1655-60.
36. Tuppin P, Ricci-Renaud P, de Peretti C, Fagot-Campagna A, Alla F, Danchin N, et al. Frequency of cardiovascular diseases and risk factors treated in France according to social deprivation and residence in an overseas territory. International Journal of Cardiology. 2014;173(3):430-5. doi: https://doi.org/10.1016/j.ijcard.2014.03.012.
37. Rosengren A, Wilhelmsen L, Orth-Gomér K. Coronary disease in relation to social support and social class in Swedish men: A 15 year follow-up in the study of men born in 1933. European heart journal. 2004;25(1):56-63. doi: 10.1016/j.ehj.2003.10.005.
38. Mookadam F, Arthur HM. Social Support and Its Relationship to Morbidity and Mortality After Acute Myocardial Infarction: Systematic Overview. Archives of Internal Medicine. 2004;164(14):1514-8. doi: 10.1001/archinte.164.14.1514.
39. Lu X, Juon H-S, He X, Dallal CM, Wang MQ, Lee S. The Association Between Perceived Stress and Hypertension Among Asian Americans: Does Social Support and Social Network Make a Difference? Journal of Community Health. 2019;44(3):451-62. doi: 10.1007/s10900-018-00612-7.
40. Compare A, Zarbo C, Manzoni GM, Castelnuovo G, Baldassari E, Bonardi A, et al. Social support, depression, and heart disease: a ten year literature review. Front Psychol. 2013;4:384-. doi: 10.3389/fpsyg.2013.00384. PubMed PMID: 23847561.
41. Xia N, Li H. Loneliness, Social Isolation, and Cardiovascular Health. Antioxidants & Redox Signaling. 2017;28(9):837-51. doi: 10.1089/ars.2017.7312.
42. Valtorta NK, Kanaan M, Gilbody S, Ronzi S, Hanratty B. Loneliness and social isolation as risk factors for coronary heart disease and stroke: systematic review and meta-analysis of longitudinal observational studies. Heart. 2016;102(13):1009-16. Epub 2016/04/20. doi: 10.1136/heartjnl-2015-308790. PubMed PMID: 27091846; PubMed Central PMCID: PMCPMC4941172.
43. Orth-Gomér K. Stress and Social Support in Cardiovascular Disease. In: Alvarenga ME, Byrne D, editors. Handbook of Psychocardiology. Singapore: Springer Singapore; 2016. p. 701-12.
44. Xue B, Head J, McMunn A. The Impact of Retirement on Cardiovascular Disease and Its Risk Factors: A Systematic Review of Longitudinal Studies. The Gerontologist. 2019. doi: 10.1093/geront/gnz062.
45. Pedron S, Maier W, Peters A, Linkohr B, Meisinger C, Rathmann W, et al. The effect of retirement on biomedical and behavioral risk factors for cardiovascular and metabolic disease. Economics & Human Biology. 2020:100893. doi: https://doi.org/10.1016/j.ehb.2020.100893.

46. Godard M. Gaining weight through retirement? Results from the SHARE survey. Journal of health economics. 2016;45:27-46.
47. Stenholm S, Solovieva S, Viikari-Juntura E, Aalto V, Kivimäki M, Vahtera J. Change in body mass index during transition to statutory retirement: an occupational cohort study. International Journal of Behavioral Nutrition and Physical Activity. 2017;14(1):85.
48. Rosengren A, Smyth A, Rangarajan S, Ramasundarahettige C, Bangdiwala SI, AlHabib KF, et al. Socioeconomic status and risk of cardiovascular disease in 20 low-income, middle-income, and high-income countries: the Prospective Urban Rural Epidemiologic (PURE) study. The Lancet Global Health. 2019;7(6):e748-e60. doi: 10.1016/S2214-109X(19)30045-2.
49. Dégano IR, Marrugat J, Grau M, Salvador-González B, Ramos R, Zamora A, et al. The association between education and cardiovascular disease incidence is mediated by hypertension, diabetes, and body mass index. Scientific reports. 2017;7(1):12370-. doi: 10.1038/s41598-017-10775-3. PubMed PMID: 28959022.
50. Schultz William M, Kelli Heval M, Lisko John C, Varghese T, Shen J, Sandesara P, et al. Socioeconomic Status and Cardiovascular Outcomes. Circulation. 2018;137(20):2166-78. doi: 10.1161/CIRCULATIONAHA.117.029652.
51. Sillars A, Celis-Morales CA, Ho FK, Petermann F, Welsh P, Iliodromiti S, et al. Association of Fitness and Grip Strength With Heart Failure: Findings From the UK Biobank Population-Based Study. Mayo Clinic Proceedings. 2019;94(11):2230-40. doi: 10.1016/j.mayocp.2019.04.041.
52. Lee M-R, Jung SM, Kim HS, Kim YB. Association of muscle strength with cardiovascular risk in Korean adults: Findings from the Korea National Health and Nutrition Examination Survey (KNHANES) VI to VII (2014–2016). Medicine (Baltimore). 2018;97(47).
53. Rein P, Saely C, Vonbank A, Zanolin D, Drexel H. Hand grip strength significantly predicts cardiovascular event risk in patients with type 2 diabetes. J Am Coll Cardiol. 2016;67(13 Supplement):2029. doi: 10.1016/S0735-1097(16)32030-7.
54. Tikkanen E, Gustafsson S, Ingelsson E. Associations of Fitness, Physical Activity, Strength, and Genetic Risk With Cardiovascular Disease. Circulation. 2018;137(24):2583-91. doi: 10.1161/CIRCULATIONAHA.117.032432.
55. Ruan Y, Guo Y, Zheng Y, Huang Z, Sun S, Kowal P, et al. Cardiovascular disease (CVD) and associated risk factors among older adults in six low-and middle-income countries: results from SAGE Wave 1. BMC public health. 2018;18(1):778. doi: 10.1186/s12889-018-5653-9.
56. Yusuf S, Hawken S, Ôunpuu S, Dans T, Avezum A, Lanas F, et al. Effect of potentially modifiable risk factors associated with myocardial infarction in 52 countries (the INTERHEART study): case-control study. The lancet. 2004;364(9438):937-52.
57. Dowd JB, Zajacova A. Does self-rated health mean the same thing across socioeconomic groups? Evidence from biomarker data. Ann Epidemiol. 2010;20(10):743-9. doi: 10.1016/j.annepidem.2010.06.007. PubMed PMID: 20816313.

Supporting information

S1 Annex. Supplementary material. The supplementary material contains detailed information on comparison of included and excluded participants, behavioral risks overtime, sensitivity analysis of multiple imputation and complete case analysis, checking for imputation model fit, comparison of models with various correlation structure, and stratified GEE analysis for Belgium and France.

Click here to access/download
Supporting Information
S1 Annex.pdf

Socioeconomic and behavioral determinants of cardiovascular diseases among older adults in Belgium and France: a longitudinal analysis from the SHARE study

Hamid Yimam Hassen[1]*, Hilde Bastiaens[1,2], Kathleen Van Royen[1,4], Steven Abrams[2,3]

[1]Department of Primary and Interdisciplinary Care, ~~College~~ Faculty of Medicine and Health Sciences, University of Antwerp, Antwerp, Belgium

[2]Global Health Institute, ~~College~~ Faculty of Medicine and Health Sciences, University of Antwerp, Antwerp, Belgium

[3]Interuniversity Institute for Biostatistics and statistical Bioinformatics, Data Science Institute, Hasselt University, Diepenbeek, Belgium

[4]Department of Communication Studies, Faculty of Social Sciences, University of Antwerp, Antwerp, Belgium

*Corresponding author

Email: Hamid.Hassen@uantwerpen.be (HYH)

Abstract

Despite advances in the healthcare system, ~~–~~cardiovascular diseases (CVDs) are still an important public health problem with disparities in the burden within and between countries. Studies among the adult population documented that socioeconomic and environmental factors play a role in the incidence and progression of CVDs. However, evidence is scarce on the socioeconomic determinants and the interplay with behavioral risks among older adults. Therefore, we identified socioeconomic and behavioral determinants of CVDs among older adults. Our sample consisted of 14,322 people aged 50 years and above from Belgium and France who responded to the waves 4, 5, 6 and/or 7 of the Survey of Health Ageing and Retirement in Europe. The effect of determinants on the occurrence of CVD was examined using a ~~g~~Generalized ~~e~~Estimating ~~e~~Equation (GEE) approach for binary longitudinal data~~models~~. The overall rate of heart attack was 8.3%, which is 7.6% in Belgium and 9.1% in France. Whereas, 2.6% and 2.3% in Belgium and France, respectively, had experienced stroke. In the multivariable GEE model, older age [AOR: 1.057, 95%CI: 1.055–1.060], living in large cities [AOR: 1.14, 95%CI: 1.07–1.18], and retirement [AOR: 1.21, 95%CI: 1.16–1.31] were associated with higher risk of CVD. Furthermore, higher level of education [AOR: 0.82, 95%CI: 0.79–0.90], upper wealth quantile [AOR: 0.82, 95%CI: 0.76–0.86] and having social support [AOR: 0.81, 95%CI: 0.77–0.84] significantly lowers the odds of having CVD. A higher hand grip strength was also significantly associated with lower risk of CVD [AOR: 0.987, 95%CI: 0.984–0.990]. This study demonstrated that older adults who do not have social support, live in big cities, belong to the lowest wealth quantile, and have a low level of education have a higher likelihood of CVD. Therefore, community-based interventions aimed at reducing cardiovascular risks need to give more emphasis to high-risk retired older adults with lower education, no social support and those who live in large cities.

Introduction

Cardiovascular diseases (CVDs) are the leading cause of morbidity and mortality worldwide and constitute a major burden on the healthcare system in all countries [1]. In 2017, an estimated 422.7 million prevalent cases, 17.9 million deaths, and 366 million disability adjusted life years (DALYs) were attributed to CVDs [2]. In Europe, nearly half of all deaths are related to CVDs so being responsible for more deaths than any other condition [3, 4]. In Belgium and France, CVDs account for 28% and 24% of all deaths respectively [5].

Despite advances in the primary and secondary prevention, there are still disparities in the CVD burden within and across countries, some segment of the population being at a higher risk [6-8]. Previously, epidemiological studies have focused on identifying and modifying individual risk factors. As a result, several health conditions and behavioral factors such as tobacco use, unhealthy diet, alcohol consumption and physical inactivity were identified to be related with CVDs. The reduction of such unhealthy lifestyles and early detection and treatment of risk factors are effective to reduce the burden. Hence, improvements have been observed in the reduction of CVD morbidities and its associated premature mortality among adults. However, many cardiovascular risks still remain as a major public health issue at different rates globally. The burden of CVDs highly varied across segments of the population, older age, the least affluent and deprived communities are disproportionately affected [9-11]. Socioeconomic and environmental factors, including income, living condition, level of education also play an important role in the development, progression and outcomes of CVDs [12-15].

As part of the efforts to narrow disparities, the World Health Organization (WHO) established a Commission on Social Determinants of Health and developed a conceptual framework for action [16]. The framework emphasizes that social determinants as well as resources to prevent illness, are not distributed randomly throughout human society and such disparities need to be

identified. Albeit some studies assessed the socioeconomic determinants of CVDs in the general population, the evidence specific to older adults is scant. Older adults have a different lifestyle and social dynamics compared to younger counterparts [17]. As age increases, the shrinkage of social networks through deaths of friends puts older people at risk of social isolation and loneliness, which might be associated with the occurrence of CVDs in those individuals [18].

Identifying the socioeconomic and behavioral factors of CVDs and evaluating the moderation effect in between could help in improving intervention strategies to reduce the burden of CVD in the older population. The EU Horizon 2020 funded project named SPICES – Scaling-up Packages of Interventions for cardiovascular diseases in Europe and Sub-Saharan Africa, aims to reduce CVD risks among vulnerable populations through risk profiling and coaching intermediate-risk participants. The current study supports the identification of target population and area of intervention among high-risk older adults in Belgium and France. Hence, in this study, we identified the socioeconomic determinants and behavioral risks of CVDs among older adults in Belgium and France, using a longitudinal data collected in the Survey of Health, Ageing and Retirement in Europe (SHARE) study. We also assessed the moderation and interplay of socioeconomic, behavioral and clinical risk factors on CVD, particularly heart attack and stroke.

Methods and materials

Study design and participants

We used the longitudinal dataset from the SHARE survey, which collects information on health status, behavioral risks, socioeconomic condition, and social networks of individuals aged 50 years and above and their partners. Details about the sampling design, methodology, and questionnaires of the SHARE survey have been presented elsewhere [19]. Methods specific to

this study are briefly summarized here. A population-based sample of the non-institutionalized population is used for this survey, with data collected via multi-stage sampling. We used data from wave 4 (2011–12), wave 5 (2013), wave 6 (2015) and wave 7 (2017) for both Belgium and France. Data from wave 3 (2008) were not included, as these data assessed the life histories of participants and did not provide the necessary information regarding health and behavioral risks. Moreover, due to the wide time interval between waves 1 (2004) and 2 (2006) on the one hand and wave 4 (2011) and subsequent waves on the other hand (five years), thereby resulting in interruption of the longitudinal nature of the data, we relied on data derived from waves 4 to 7.

Participants fulfilling the following criteria were included to the analysis: i) aged 50 years or above and ii) having information on the main outcome variables, heart attack, stroke or atherosclerosis at least in one wave. Data were not analyzed for participants i) < 50 years of age and ii) people who were reported as having CVD during enrollment, and iii) with no CVD information at~~during~~ enrollment~~ due to missing values~~. Overall, 7,914 adults in Belgium and 6,408 in France participated in at least one wave of the survey and were eligible, resulting in a total number of person-observations of 20,555 and 16,305 for Belgium and France, respectively.

Outcome and exposure assessment

Cardiovascular diseases

The presence or absence of cardiovascular diseases was measured in the SHARE study using the question ‘*Has a doctor told you that you had/Do you currently have any of the conditions on this card?*’. The possible answers were: yes or no; if yes, the time of first diagnosis was asked. In this study, we defined a person as having CVD if he/she has at least one of the

following problems, or both; i) a heart attack including myocardial infarction or coronary thrombosis; or ii) a stroke or cerebrovascular disease diagnosed between waves.

Socioeconomic determinants

Socioeconomic variables, including age, sex, household income, retirement status, marital status, educational status, and social support were collected in the SHARE study. In our study, we categorized marital status as a dichotomous variable as living with a partner or living alone, where the latter included those who were widowed, divorced or never married. Level of education was assessed with the International Standard Classification of Education (ISCED) 1997 [20], and was categorized as low (level 0-2), medium (levels 3 and 4), and high (levels 5 and 6). We categorized wealth using three quantiles for each country into upper, middle and lower one-third. We categorized living areas in three groups: i) large town or city, ii) small town, and iii) rural or village. Social support was measured using two questions: (1) ‘*In the past twelve months, has any family member from outside the household, friend, or neighbor given you any type of support?*’ and, if ‘yes’, (2) ‘*Is there someone living in this household who has helped you regularly during the past twelve months*?’. We dichotomized this information by defining social support as having positive responses on both of the above questions, and no social support otherwise.

Behavioral determinants

Smoking status in the SHARE survey was assessed by asking ‘*Do you smoke at the present time?’*. Regular alcohol consumption was assessed with the question ‘*In the last three months, how often did you have six or more units of alcoholic beverages on one occasion?*’. The possible response options were: i) daily or almost daily, ii) 5 or 6 days a week, iii) 3 or 4 days a week, iv) once or twice a week, v) once or twice a month, vi) less than once a month and vii) not at all in the last 3 months. We dichotomized this variable into regular alcohol consumption

(i-iv) and no regular alcohol consumption (v-vii). The frequency of engaging in vigorous and moderate physical activity (PA) was assessed asking: '*How often do you engage in vigorous/moderate PA, such as sports, heavy housework, or a job that involves physical labor?*' The possible responses were: i) more than once a week, ii) once a week, iii) once to three times a month, and iv) hardly ever or never. We dichotomized this variable into regular PA (i and ii), and no regular PA (iii and iv). Fruit and vegetable intake was assessed by asking: '*In a regular week, how often do you have a serving of fruit and vegetables?'*. The possible responses were: i) every day, ii) three to six times a week, iii) twice a week, iv) once a week, v) less than once a week. We dichotomized into adequate (i) and inadequate intake (ii-v).

Physical measurements and clinical characteristics

We computed body mass index (BMI) using the self-reported values of body weight and height in each wave. Hand-grip strength was measured by trained interviewers using a handheld dynamometer. Self-reported long-term illnesses were assessed with the question '*Do you have any long-term health problems, illness, disability or infirmity such as chronic respiratory, kidney disease, cancer, etc.?*'. We categorized comorbidities into; i) no comorbidity, ii) one comorbidity, and iii) two and more comorbidities. Depression status was assessed using a EURO-12, a 12-item geriatric depression scale and those who scored below 4 were considered as not having depression and those above 4 as having depression. Moreover, whether the participant ever had CVD risks, including high blood pressure or hypertension; high blood cholesterol; and diabetes and/or high blood sugar were assessed.

Formatted: Space Before: 12 pt

Statistical analysis

Descriptive statistics including absolute and relative frequencies for categorical variables, mean with Standard Deviation (SD) or median with Inter Quartile Range (IQR) for

continuous variables were employed to summarize the characteristics of participants. We compared~~ the~~ socioeconomic characteristics between ~~thos~~individualse included int~~o~~ the analysis (n = 14,322) ~~–~~and those excluded as a result of missing CVD outcome values at enrollment (n = 117)~~due to exclusion criteria~~, using two sample t-tests and chi-square tests for numerical and categorical variables, respectively. The results show that individuals who were included are comparable with the excluded ones in terms of socioeconomic characteristics (*table 1 in S1 Appendix*). To estimate the population averaged effect of socioeconomic, behavioral and physical determinants on the occurrence of CVD, we developed two independent marginal models using multivariable ~~g~~Generalized ~~e~~Estimating ~~e~~Equations (GEEs) with the binomial distribution and logit link function [21]. ~~The~~ GEE accounts for within-subject association and~~ helps~~ allows to estimate the between-subject effects of the relationships of determinants with the occurrence of CVD over the observation period. The unequal selection probabilities and unequal numbers of follow-up interviews among study participants was taken into consideration in the GEE analysis using individual survey weights. We assumed that the within-subject association among the vectors of repeated outcomes would have an exchangeable working correlation structure. Albeit other correlation structures were considered, ~~the best~~ goodness-of-fit indices were found under~~in~~ the exchangeable correlation structure (*table 3~~2~~ in S1 Appendix*). As a goodness-of-fit indices, we used the quasi-likelihood under the Independence Model Criterion (QIC), the Wald $\chi 2$ test, and we performed a residual analysis to assess the presence of outliers and their random distribution [22, 23]. To compare the models, we used both the QIC and the Wald-test between the null, non-adjusted model and the adjusted models. Hence, the model with an exchangeable correlation structure was selected as it provided the smallest QIC.

~~To examine the overall mediation effect of behavioral, physical and clinical characteristics on the association between socioeconomic factors and CVD, we developed four GEE models and~~

Formatted: Not Highlight

compared the change in the effect sizes. *Mode 1* contains socioeconomic determinants including age, sex, living area, wealth quantile, educational level, retirement, marital status, and social support. In *model 2*, we used all variables in model 1 and behavioral determinants, including physical activity, fruit and vegetable intake, smoking and alcohol consumption. In *model 3*, variables in model 2 and additional clinical and physical measurements (BMI, grip strength, presence of comorbidity, and depression) were included. In *model 4*, we used variables in model 1 and immediate risk factors, including hypertension, high blood cholesterol and high blood sugar (*table 6 in S1 Appendix*). To evaluate whether the determinants vary between Belgium and France, we also consideredperformed a stratified analysis fittinged a model on a separate dataset for each country separately, however, there was no significant difference between countries in terms of covariate effectsvariation and the results are shown in the supplement (*table 7 and 8 in annex 1*). We performed the GEE analyses using the '**geepack**' package of the free statistical software, R version 3.6.1 [24].

Formatted: Font: Italic

Formatted: Not Highlight

Formatted: Font: Italic

The percentage of missing values across all the variables varied between 0 and 37.6%. Missing results were imputed for all missing variables evaluated in the GEE model. Assuming the missing data to be missing at random (MAR), we performed a Mmultivariate Iimputation by Cchained Eequations (MICE) using the '**mice'** package in R [25]. For more details regarding the MICE approach, we refer the reader to Van Buuren (2018) [27]. We imputed the dataset to have M = 100 complete datasets using all the variables included intoo the model and other some additional additional auxiliary variables (for more details regardingdetailed description on the imputation model , the reader is referred to theis available in the supplementary material). For more details regarding the MICE approach we used, we refer the reader to a book by Van Buuren [26]. The parameters of interest were estimated in each imputed dataset separately, and combined using Rubin's rules [27]. We performed a sensitivity analysis to compare the results obtained from a multiple imputation approach with those from a complete

case analysis. The results were ~~relatively~~ comparable between the two approaches, except ~~for a few variation~~ in terms of precision of the estimated model parameters~~, suggesting that missingness had a minimal impact on the precision of results~~. More specifically, m~~M~~ultiple imputation was found to lead to more precise estimates which can be seen from~~more efficient as can be seen from~~ the shorter confidence intervals (*table 4~~3~~ and 5~~4~~ in S1 Appendix*). We assessed the distributional similarity of the observed and imputed observations using summary statistics and showed that the distributions of the imputed and observed values ~~were~~ are ~~relatively~~ comparable~~.~~ (*table 6~~5~~ in S1 Appendix*).

Ethics statement

We used publicly available data (available via the website http://www.share-project.org/data-documentation/data-documentation-tool.html) for a secondary data analysis. SHARE underwent a review of ethical standards by the University of Mannheim's internal review board. Ethical considerations including the written informed consent has been taken care of by another institution. Details on the conduct of the study including the ethical approval can be found elsewhere [28].

Formatted: Space Before: 12 pt

Formatted: Font color: Auto

Results

Socioeconomic characteristics participants

Table 1 displays the socioeconomic characteristics of participants at their first enrollment. The mean age of participants was 64.4 (SD: 10.9) and 65.6 (SD: 10.9) in Belgium and France, respectively. Overall, 55.7% were females and 41.8% had low level of education. Majorities (67.0%) were living with their partners and one-third (33.1%) were living in large towns or cities. More than half (55.4%) were retired and nearly one-third (31.8%) had regular social support from family members or any other person. The median household annual net income was 32.6 and 28.6 thousand Euro in Belgium and France, respectively.

Table 1. Socioeconomic characteristics of study participants during enrollment to interview stratified by country (n=14,322).

Participant characteristics	Total	Belgium	France
**Age** *(years)*	64.9 (10.9)	64.4 (10.9)	65.6 (10.9)
**Sex** *(Ffemale)*	7975 (55.7)	4340 (54.8)	3635 (56.7)
**Level of education** [a] (n=14,130)			
Low	5902 (41.8)	3135 (40.0)	2767 (44.0)
Medium	4327 (30.6)	2121 (27.0)	2206 (35.0)
High	3901 (27.6)	2579 (32.9)	1322 (21.0)
**Marital status** (n=14,250)			
With partner	9547 (67.0)	5338 (67.6)	4209 (66.2)
Alone	4703 (33.0)	2553 (32.4)	2150 (33.8)
**Living area** (n=13,793)			
Rural	4965 (36.0)	2088 (27.5)	2877 (46.5)
Small town	4263 (30.9)	2632 (34.6)	1631 (26.3)
Large town or city	4565 (33.1)	1631 (26.3)	1683 (27.2)
**Family size (no of persons)**			
One	3869 (27.0)	2071 (26.2)	1798 (28.1)
Two	7989 (55.8)	4326 (54.7)	3663 (57.2)
Three	1493 (10.4)	923 (11.7)	570 (8.9)
Four and above	971 (6.8)	594 (7.5)	377 (5.9)
**Estimated household income** (€) (median(IQR)) (n=14,109)	30463.22 (31030.7)	32640.3 (35174.8)	28621.0 (26953.5)
Lower	4404 (31.2)	2377 (30.6)	2027 (32.0)
Middle	4452 (31.6)	2421 (31.1)	2031 (32.1)
Upper	5253 (37.2)	2984 (38.3)	2269 (35.9)
**Social support** (n=11,275)			
Yes	3589 (31.8)	2166 (34.7)	1423 (28.3)
No	7686 (68.2)	4083 (65.3)	3603 (71.7)
**Retirement** (n=13,994)			
Yes	7939 (56.7)	3971 (51.7)	3968 (62.9)
No	6055 (43.3)	3717 (48.3)	2338 (37.1)

a- Based on ISCED 1997 (level 0-2)

a-

Formatted: Indent: Left: 0.74", No bullets or numbering

Behavioral, physical measurements and other health related characteristics

The trend of behavioral, physical and other health related characteristics of participants at each wave of data collection is presented in detail in the supplementary material (*table 21 in S1 Appendix*). Overall, 87.6% of participants reported adequate fruit and vegetable intake, i.e., at least one serving per day and the trend was consistent from 2011 to 2017. Above half (50.9%) hardly ever or never involved in vigorous physical activities, whereas, 65.6% reported that they

performed moderate physical activities more than once a week. Similarly, the frequency of vigorous and moderate physical activity remains relatively constant across time. Overall, only 7.8% have a history of regular alcohol consumption within 6 months prior to data collection. Overall, one-fifth (19.9%) were smokers at the time of each interview and the proportion was highest in 2017 (29.2%). The rate of obesity is relatively consistent throughout the observation period, ranging 18.6% (2011) to 20.7% (2015). The overall mean of hand grip strength was 33.6 (SD: 11.8) and it was consistent across the waves. The rate of depression ranged from 29.9% in 2017 to 31.5% in 2015, with an overall rate of 30.9%.

The prevalence of cardiovascular risks

Table 2 summarizes the prevalence of CVD risks in Belgium and France. One-third (33.2%) and 31.0% of older adults in Belgium and France, respectively, had a history of hypertension at least once in their life time. The prevalence of high blood cholesterol in Belgium (30.6%) is higher than in France (23.3%). Whereas, the prevalence of high blood sugar in Belgium (10.8%) is nearly equal to the prevalence in France (10.9%).

Table 2. The prevalence of cardiovascular risks stratified by country (n=14276)

Cardiovascular risks	Total n(%)	Belgium n(%)	France n(%)
Ever had hypertension *(Yes)*	4596 (32.2)	2617 (33.2)	1979 (31.0)
Ever had high blood cholesterol *(Yes)*	3898 (27.3)	2410 (30.6)	1488 (23.3)
Ever had high blood sugar *(Yes)*	1546 (10.8)	848 (10.8)	698 (10.9)
Total	14276	7888	6388

The occurrence of cardiovascular events

The overall rate of heart attack was 8.3%, which is 7.6% in Belgium and 9.1% in France. The rate of stroke was 2.6% and 2.3% in Belgium and France, respectively. Throughout the observation period, the overall rate of CVD was 9.5% and 10.7% in Belgium and France, respectively. (Table 3)

Table 3. The occurrence of cardiovascular diseases stratified by country (n=36,781 person observation points; Belgium=20,516 and France=16,265).

CVD events	Overall percentage	Belgium N (%)	France N (%)
Heart attack	8.3	1563 (7.6)	1483 (9.1)
Stroke	2.4	526 (2.6)	369 (2.3)
Heart attack or stroke	10.0	1,954 (9.5)	1,738 (10.7)

Socioeconomic determinants of cardiovascular diseases among older adults

In the multivariable GEE model, the odds of having CVD was 5.7% higher for a one year increase in age [AOR: 1.057, 95%CI: 1.055–1.060]. The risk of CVD is significantly higher among those who were living in large cities [AOR: 1.14, 95%CI: 1.07–1.18] than those living in rural areas. Similarly, retired older adults showed a higher odds of CVD than those who were not retired [AOR: 1.21, 95%CI: 1.16–1.31]. Being female [AOR: 0.54, 95%CI: 0.51–0.56], high level of education [AOR: 0.82, 95%CI: 0.79–0.90], higher income [AOR: 0.82, 95%CI: 0.76–0.86] and social support [AOR: 0.81, 95%CI: 0.77–0.84] imply a significantly lower risk of CVD as compared to their respective reference categories. (Table 4)

Table 4. Multivariable~~te~~ GEE models estimating the effect of socioeconomic characteristics on CVD among older adults in Belgium and France.

Variables	Percent with outcome	COR [95%CI]	AOR [95%CI]
**Age**	*Cont.*	1.063 [1.059–1.067]***	1.057 [1.055–1.060]***
**Sex**			
Male	12.6	1	1
Female	8.0	0.60 [0.55–0.65]***	0.54 [0.51–0.56]***
**Living area**			
Rural	9.6	1	1
Small	10.4	1.08 [0.99–1.18]	1.08 [0.99–1.14]
Large city/town	10.5	1.14 [1.01–1.22]*	1.14 [1.07–1.18]*
**Level of education**			
Primary	12.7	1	1
Secondary	8.6	0.64 [0.58–0.72]**	0.91 [0.86–0.97]*
Higher	7.8	0.58 [0.52–0.65]***	0.82 [0.79–0.90]**
**Marital status**			
Partner	9.2	1	1
Alone	11.5	1.30 [1.19–1.41]*	1.04 [0.99–1.10]
**Net income**			
Lower	12.1	1	1
Middle	10.3	0.86 [0.79–0.94]*	0.99 [0.94–1.03]
Upper	7.0	0.60 [0.55–0.67]***	0.82 [0.76–0.86]**
**Social support**			
No	12.2	1	1
Yes	7.0	0.63 [0.58–0.69]***	0.81 [0.77–0.84]***
**Retirement**			
No	5.8	1	1
Yes	13.0	2.10 [1.92–2.28]***	1.21 [1.16–1.31]**

** p–value<0.05; ** p<0.01; *** p <0.001*
AOR: Adjusted odds ratio; COR: Crude odds ratio;
Multivariate multiple imputations were performed (n=36,860)

- *Interaction of age and level of education with living area, income and social support was assessed but statistically not significant.*

Behavioral and physical determinants of cardiovascular diseases among older adults

In the multivariable~~te~~ GEE model (Table 5), on average the odds of CVD is 31% lower for those who do regular physical activity than those who do not [AOR: 0.69, 95%CI: 0.64–0.73].

Similarly, adequate fruit and vegetable intake is also associated with lower odds of CVD among older adults [AOR: 0.93, 95%CI: 0.87–0.99]. The odds of having CVD is 1.5 times higher for those who are obese than for those not obese [AOR: 1.49, 95%CI: 1.44–1.55]. Those who have one comorbidity and two or more multimorbidity are 2.2 and 4.5 times respectively, higher likelihood of having CVD than those with no comorbidity. The odds of having CVD is also 1.3 times higher for those who have depression than their counterparts [AOR: 1.27, 95%CI: 1.21–1.33]. Moreover, hand grip strength is significantly associated with lower CVD [AOR: 0.987, 95%CI: 0.984–0.990].

Table 5. Multivariable GEE model estimating the effect of behavioral and physical determinants of cardiovascular diseases among older adults in Belgium and France

**Variables**	**Percent with outcome**	**COR [95%CI]**	**AOR [95%CI]**
**Age**	*Cont.*	1.063 [1.059–1.067]***	1.045 [1.042–1.048]***
**Sex** *(female)*	8.0	0.60 [0.55–0.65]***	0.42 [0.39–0.45]***
**Physical activity**			
No regular PA	12.6	1	1
Regular PA	6.1	0.55 [0.51–0.60]***	0.69 [0.64–0.73]**
**Fruit and vegetable intake**			
Not adequate	10.2	1	1
Adequate	9.3	0.93 [0.82–1.06]	0.93 [0.87–0.99]*
Smoking			
No	9.1	1	1
Yes	7.9	1.16 [1.02–1.31]*	1.19 [1.13–1.25]*
**Regular alcohol consumption**			
No	11.4	1	1
Yes	10.8	0.99 [0.80–1.21]	1.04 [0.96–1.13]
**BMI**			
Normal	8.2	1	1
Overweight	10.0	1.20 [1.10–1.31]***	1.08 [1.03–1.14]*
Obesity	13.9	1.67 [1.50–1.85]***	1.49 [1.44–1.55]***
**Chronic comorbidities**			
No comorbidity	2.1	1	1
One comorbidity	8.1	2.84 [2.52–3.21]***	2.23 [2.10–2.37]***
>=2 comorbidities	21.9	6.98 [6.20–7.87]***	4.54 [4.27–4.83]***
**Grip strength**	*cont.*	0.984 [0.980–0.988]***	0.987 [0.984–0.990]*
**Depression**			
No	8.2	1	1
Yes	13.0	1.51 [1.40–1.63]***	1.27 [1.21–1.33]**

* *p–value<0.05; ** p<0.01; *** p <0.001*

AOR: Adjusted odds ratio; COR: Crude odds ratio; BMI: Body Mass Index
Multivariate multiple imputations were performed (n=36,860).

- *Interaction of physical activity level with fruit and vegetable intake and smoking with alcohol consumption was examined but statistically not significant*

~~The mediation analysis shows the AORs of socioeconomic determinants attenuated when the behavioral determinants were included into the model, indicating a possible mediating effect. The excess odds of CVD due to living in large cities decreased by 27% (14.2% vs 10.3%) and retirement by 48% (21.1% vs 11.0%). The decrease in the odds of CVD due to high level of education lowered by 15.3 % (17.6% vs 14.9%); higher income by 33.2% (18.4% vs 12.3%); and social support by 36.9% (19.5% vs 12.3%). Moreover, adding physical parameters and other clinical characteristics to the model showed a decrease in the AORs. Similarly, fitting other risk factors including hypertension, high blood cholesterol and high blood sugar to the model showed a decrease in the AORs for socioeconomic determinants, showing a possible mediating effect. However, as we did not assess the individual mediation effect, we cannot estimate the direct and indirect effect of each determinant on CVD. Details of the mediation analysis are available in the supplement (*table 6 in S1 Appendix*).~~

Discussion

Based on a large sample of adults aged 50 years and above in Belgium and France, this study aimed to identify the socioeconomic, behavioral and physical determinants of cardiovascular diseases and the interplay in between longitudinally. Whilst several studies evidenced the role of socioeconomic factors on CVD among adults, the role of these factors on older people were not well documented. This study showed that socioeconomic characteristics, including having social support, level of education, living area, retirement and income level were independent determinants of CVD among older adults.

After adjustment for the socioeconomic~~demographic~~ and other related variables, the risk of having CVD was significantly higher among older adults who live in large cities than those

who live in villages. A lower green area in urban settings could be a possible reason as indicated by Seo S. and colleagues who found adults living in areas with greater amounts of green space found to have a lower risk of CVD [29]. Consistently, various studies showed that exposure to rural green space is associated with a reduction in indicators of CVD risk factors compared to urban streets [30-34]. The higher risk of CVD in the most deprived groups is more pronounced in urban areas with low amounts of green space coverage [35, 36]. Furthermore, the higher prevalence of smoking, physical inactivity, and unhealthy diet in urban areas could also be the possible reasons for higher risk of CVD. Thus, interventions aimed at reduction of CVD risks need to integrate with other sectors for optimal intervention effect.

In our study, older adults with regular social support from family or anyone else have a lower risk of CVD. This finding is similar to a study by Rosengren AL. *et al,* which indicate low social support is associated with coronary heart diseases [37]. Several studies also documented the role of social support in CVD incidence and mortality [38-40]. Both objective social isolation and the subjective perception of being isolated have been shown to be associated with a higher rate of CVD [41, 42]. Orth-Gomer explained the psychological mechanisms of social support leading to CVD morbidity and mortality [43]. Therefore, it is essential to elucidate the molecular mechanisms of loneliness leading to CVDs. Moreover, community based CVD prevention strategies need to consider the role of social support to prevent the detrimental effects of isolation.

The present study showed, retired older adults have a higher risk of CVD after adjusted for age, sex and other socioeconomic characteristics. A recent systematic review indicated the impact of retirement on the rate of CVDs and risk factors varies across countries, in which studies in the European countries showed a detrimental effect of retirement on CVDs [44]. Pedron and colleagues identified male and low-educated retirees as potential high-risk groups for worsening CVD risk factors after retirement [45]. Retirement has been linked to increased

leisure time activities but, it may reduce transport and work related activities. The effect of retirement also varied with the type of job a person retired from. A study by Godard and associates showed retirement caused an increase in the likelihood of being obese among men retiring from strenuous jobs [46]. Our mediation analysis also indicates nearly half of the effect of retirement is mediated by behavioral risks. On the other hand Stenholm and colleagues indicated retirement was associated with slight weight loss in men retiring from sedentary jobs [47]. Hence, policies concerning the retirement age need to focus on ensuring whether they are suited to individuals and contexts.

In this study, higher income and higher level of education are associated with lower risk of CVDs. A multi-country study showed cardiovascular events were more common among those with low levels of education [48]. Dégano and her colleagues also indicated the rate of CVD events is 50% lower for those with university education compared to primary or lower education [49] The variation in CVD incidence and mortality based on the socioeconomic status has also been documented elsewhere [12, 14, 15, 50].

Various physical factors were also found significant in our study. Grip strength is associated with CVD after adjusting for socioeconomicdemographic and behavioral factors. Previous studies showed the association of hand grip strength with heart failure and cardiovascular risks such as high blood pressure, high blood sugar and lipid levels [51, 52]. Hand grip strength in patients with type 2 diabetes is inversely associated with CVD independently from well-established cardiovascular risks [53]. A prospective study from the UK also showed hand grip strength has inverse associations with incidence of cardiovascular events [54]. This implies hand grip strength could be a valuable CVD risk assessment tool for older adults in combination with the already available indicators.

Our findings also showed behavioral factors, including physical inactivity, inadequate fruit and vegetable intake, obesity, smoking and depression are associated with higher risk of cardiovascular events, as it is evidenced from several studies [55, 56], indicating older adults are not an exception in this regard.

The findings from this study need to be interpreted in the context of the following limitations. First, we used the existing measures from the SHARE ~~survey~~ study and were not able to include more measures of determinants that might~~ay~~ be more appropriate to estimate the effect of behavioral determinants on CVD. For instance, we could not explore the amount of serving of fruit and vegetable intake, the duration and intensity of vigorous and moderate physical activity level, and the amount of alcohol consumption. Secondly, the measurement of socioeconomic characteristics, CVDs and risks was self-reported. We did not assess it using physical or laboratory measurements. Participants in low socioeconomic status (SES) could under-report their CVD status and risks [57], which might nullify the effect size of SES on CVD. This indicates the effect could even be more than what we found. Further studies with a more objective assessment of CVDs and risks might provide a larger effect size. Nevertheless, ~~the use of~~SHARE used cards with a list of disease conditions to probe participants, which could minimize the reporting bias. ~~-~~Thirdly, as our aim was not to identify a single determinant, we did not perform a comprehensive mediation analysis for each determinant-mediator-outcome relationship. ~~However, we roughly summarized the change in effect sizes when including behavioral and physical determinants to the model~~We recommend future studies to identify independent socioeconomic and behavioral determinants of CVDs with extensive mediation analysis.

Conclusions

This study addressed several aspects of cardiovascular disease prevention areas among older adults. Our research demonstrated that older adults who are retired, do not have social support, live in big cities, belong to the lowest wealth quantile and have a low educational level have a higher likelihood of CVD. It also showed that behavioral risks are prevalent in older adults living in Belgium and France and associated with an increase in CVD as documented from our study and several other studies. Physical factors such as a better hand grip strength is associated with lower incidence of CVD. The findings from this study underlined the fact that socioeconomic disparities affect the occurrence of CVDs in older adults. Therefore, community based interventions aimed at improving cardiovascular risks need to give more emphasis to high-risk older adults to get optimal benefit from the interventions. Older adults who live in large cities with no social support need to gain more emphasis to halt the continued problem of CVD and associated premature mortality.

Formatted: Line spacing: Double

References

1. Roth GA, Johnson C, Abajobir A, Abd-Allah F, Abera SF, Abyu G, et al. Global, Regional, and National Burden of Cardiovascular Diseases for 10 Causes, 1990 to 2015. J Am Coll Cardiol. 2017;70(1):1-25. Epub 2017/05/17. doi: 10.1016/j.jacc.2017.04.052. PubMed PMID: 28527533.
2. WHO. A global factsheet on cardiovascular diseases 2017 [Jan 20, 2020]. Available from: https://www.who.int/news-room/fact-sheets/detail/cardiovascular-diseases-(cvds).
3. Townsend N, Wilson L, Bhatnagar P, Wickramasinghe K, Rayner M, Nichols M. Cardiovascular disease in Europe: epidemiological update 2016. European heart journal. 2016;37(42):3232-45. Epub 2016/08/16. doi: 10.1093/eurheartj/ehw334. PubMed PMID: 27523477.
4. WHO regional office for Europe. Cardiovascular Diseases; Data and Statistics 2018 [Feb 1, 2020]. Available from: http://www.euro.who.int/en/health-topics/noncommunicable-diseases/cardiovascular-diseases/data-and-statistics.
5. EUROstat. Causes of death — diseases of the circulatory system, residents 2016. Available from: https://ec.europa.eu/eurostat/statistics-explained/index.php?title=Causes_of_death_statistics.
6. Marmot M, Wilkinson R. Social determinants of health: OUP Oxford; 2005.
7. Menotti A, Puddu PE, Lanti M, Kromhout D, Blackburn H, Nissinen A. Twenty-five-year coronary mortality trends in the seven countries study using the accelerated failure time model. Eur J Epidemiol. 2003;18(2):113-22. Epub 2003/05/08. doi: 10.1023/a:1023092415353. PubMed PMID: 12733832.

8. Chow CK, Lock K, Teo K, Subramanian SV, McKee M, Yusuf S. Environmental and societal influences acting on cardiovascular risk factors and disease at a population level: a review. Int J Epidemiol. 2009;38(6):1580-94. Epub 2009/03/06. doi: 10.1093/ije/dyn258. PubMed PMID: 19261658; PubMed Central PMCID: PMCPMC2786248.
9. North BJ, Sinclair DA. The intersection between aging and cardiovascular disease. Circ Res. 2012;110(8):1097-108. Epub 2012/04/14. doi: 10.1161/circresaha.111.246876. PubMed PMID: 22499900; PubMed Central PMCID: PMCPMC3366686.
10. Yazdanyar A, Newman AB. The burden of cardiovascular disease in the elderly: morbidity, mortality, and costs. Clin Geriatr Med. 2009;25(4):563-77, vii. Epub 2009/12/01. doi: 10.1016/j.cger.2009.07.007. PubMed PMID: 19944261; PubMed Central PMCID: PMCPMC2797320.
11. Benjamin Emelia J, Muntner P, Alonso A, Bittencourt Marcio S, Callaway Clifton W, Carson April P, et al. Heart Disease and Stroke Statistics—2019 Update: A Report From the American Heart Association. Circulation. 2019;139(10):e56-e528. doi: 10.1161/CIR.0000000000000659.
12. Havranek Edward P, Mujahid Mahasin S, Barr Donald A, Blair Irene V, Cohen Meryl S, Cruz-Flores S, et al. Social Determinants of Risk and Outcomes for Cardiovascular Disease. Circulation. 2015;132(9):873-98. doi: 10.1161/CIR.0000000000000228.
13. Psaltopoulou T, Hatzis G, Papageorgiou N, Androulakis E, Briasoulis A, Tousoulis D. Socioeconomic status and risk factors for cardiovascular disease: Impact of dietary mediators. Hellenic Journal of Cardiology. 2017;58(1):32-42. doi: https://doi.org/10.1016/j.hjc.2017.01.022.
14. Lemstra M, Rogers M, Moraros J. Income and heart disease: Neglected risk factor. Can Fam Physician. 2015;61(8):698-704. PubMed PMID: 26836056.
15. Lewis MW, Khodneva Y, Redmond N, Durant RW, Judd SE, Wilkinson LL, et al. The impact of the combination of income and education on the incidence of coronary heart disease in the prospective Reasons for Geographic and Racial Differences in Stroke (REGARDS) cohort study. BMC public health. 2015;15(1):1312. doi: 10.1186/s12889-015-2630-4.
16. Solar O, Irwin A. A conceptual framework for action on the social determinants of health. Social Determinants of Health Discussion Paper 2 (Policy and Practice). Geneva: World Health Organization, 2010.
17. Smedley BD, Syme SL. Institute of Medicine (US), Committee on Capitalizing on Social Science and Behavioral Research to Improve the Public's Health, 2000. Promoting health: intervention strategies from social and behavioral research, xiv National Academy Pr, Washington, DC.
18. Leigh-Hunt N, Bagguley D, Bash K, Turner V, Turnbull S, Valtorta N, et al. An overview of systematic reviews on the public health consequences of social isolation and loneliness. Public Health. 2017;152:157-71. doi: https://doi.org/10.1016/j.puhe.2017.07.035.
19. Survey of Health, Ageing and Retirement in Europe. 2017. Available at: http://www.share-project.org (20 February 2020, date last accessed).
20. UNESCO. International Standard Classification of Education 1997. [cited 2020 May 8]. Available from: http://www.unesco.org/education/information/nfsunesco/doc/isced_1997.htm.
21. Zeger SL, Liang K-Y, Albert PS. Models for Longitudinal Data: A Generalized Estimating Equation Approach. Biometrics. 1988;44(4):1049-60. doi: 10.2307/2531734.
22. Pan W. Akaike's Information Criterion in Generalized Estimating Equations. Biometrics. 2001;57(1):120-5. doi: 10.1111/j.0006-341X.2001.00120.x.
23. Hardin JW, Hilbe JM. Generalized Estimating Equations: Chapman & Hall/CRC. Boca Raton, Florida. 2003.
24. Halekoh U, Højsgaard S, Yan J. The R package geepack for generalized estimating equations. Journal of Statistical Software. 2006;15(2):1-11.
25. Van Buuren S, Groothuis-Oudshoorn K. mice: multivariate imputation by chained equations in RJ Stat. Softw. 45, 1–67. 2011.
26. Van Buuren S. Flexible imputation of missing data: CRC press; 2018.
27. Rubin DB. Multiple Imputation for Nonresponse in Surveys: John Wiley & Sons; 2009.
28. Alcser KH, Benson G, Börsch-Supan A, Brugiavini A, Christelis D, Croda E, et al. The survey of health, aging, and retirement in Europe—Methodology. Mannheim Mannheim Research Institute for the Economics of Aging (MEA). 2005.

29. Seo S, Choi S, Kim K, Kim SM, Park SM. Association between urban green space and the risk of cardiovascular disease: A longitudinal study in seven Korean metropolitan areas. Environment International. 2019;125:51-7. doi: https://doi.org/10.1016/j.envint.2019.01.038.
30. Lanki T, Siponen T, Ojala A, Korpela K, Pennanen A, Tiittanen P, et al. Acute effects of visits to urban green environments on cardiovascular physiology in women: A field experiment. Environmental research. 2017;159:176-85.
31. Grazuleviciene R, Vencloviene J, Kubilius R, Grizas V, Dedele A, Grazulevicius T, et al. The effect of park and urban environments on coronary artery disease patients: a randomized trial. BioMed research international. 2015;2015.
32. Kardan O, Gozdyra P, Misic B, Moola F, Palmer LJ, Paus T, et al. Neighborhood greenspace and health in a large urban center. Scientific reports. 2015;5:11610.
33. Nowicki GJ, Ślusarska B, Piasecka H, Bartoszek A, Kocka K, Deluga A. The Status of Cardiovascular Health in Rural and Urban Areas of Janów Lubelski District in Eastern Poland: A Population-Based Study. Int J Environ Res Public Health. 2018;15(11):2388. doi: 10.3390/ijerph15112388. PubMed PMID: 30373289.
34. Sarrafzadegan N, Talaei M, Kelishadi R, Toghianifar N, Sadeghi M, Oveisgharan S, et al. The influence of gender and place of residence on cardiovascular diseases and their risk factors. The Isfahan cohort study. Saudi Med J. 2012;33(5):533-40. Epub 2012/05/17. PubMed PMID: 22588815.
35. Mitchell R, Popham F. Effect of exposure to natural environment on health inequalities: an observational population study. The lancet. 2008;372(9650):1655-60.
36. Tuppin P, Ricci-Renaud P, de Peretti C, Fagot-Campagna A, Alla F, Danchin N, et al. Frequency of cardiovascular diseases and risk factors treated in France according to social deprivation and residence in an overseas territory. International Journal of Cardiology. 2014;173(3):430-5. doi: https://doi.org/10.1016/j.ijcard.2014.03.012.
37. Rosengren A, Wilhelmsen L, Orth-Gomér K. Coronary disease in relation to social support and social class in Swedish men: A 15 year follow-up in the study of men born in 1933. European heart journal. 2004;25(1):56-63. doi: 10.1016/j.ehj.2003.10.005.
38. Mookadam F, Arthur HM. Social Support and Its Relationship to Morbidity and Mortality After Acute Myocardial Infarction: Systematic Overview. Archives of Internal Medicine. 2004;164(14):1514-8. doi: 10.1001/archinte.164.14.1514.
39. Lu X, Juon H-S, He X, Dallal CM, Wang MQ, Lee S. The Association Between Perceived Stress and Hypertension Among Asian Americans: Does Social Support and Social Network Make a Difference? Journal of Community Health. 2019;44(3):451-62. doi: 10.1007/s10900-018-00612-7.
40. Compare A, Zarbo C, Manzoni GM, Castelnuovo G, Baldassari E, Bonardi A, et al. Social support, depression, and heart disease: a ten year literature review. Front Psychol. 2013;4:384-. doi: 10.3389/fpsyg.2013.00384. PubMed PMID: 23847561.
41. Xia N, Li H. Loneliness, Social Isolation, and Cardiovascular Health. Antioxidants & Redox Signaling. 2017;28(9):837-51. doi: 10.1089/ars.2017.7312.
42. Valtorta NK, Kanaan M, Gilbody S, Ronzi S, Hanratty B. Loneliness and social isolation as risk factors for coronary heart disease and stroke: systematic review and meta-analysis of longitudinal observational studies. Heart. 2016;102(13):1009-16. Epub 2016/04/20. doi: 10.1136/heartjnl-2015-308790. PubMed PMID: 27091846; PubMed Central PMCID: PMCPMC4941172.
43. Orth-Gomér K. Stress and Social Support in Cardiovascular Disease. In: Alvarenga ME, Byrne D, editors. Handbook of Psychocardiology. Singapore: Springer Singapore; 2016. p. 701-12.
44. Xue B, Head J, McMunn A. The Impact of Retirement on Cardiovascular Disease and Its Risk Factors: A Systematic Review of Longitudinal Studies. The Gerontologist. 2019. doi: 10.1093/geront/gnz062.
45. Pedron S, Maier W, Peters A, Linkohr B, Meisinger C, Rathmann W, et al. The effect of retirement on biomedical and behavioral risk factors for cardiovascular and metabolic disease. Economics & Human Biology. 2020:100893. doi: https://doi.org/10.1016/j.ehb.2020.100893.
46. Godard M. Gaining weight through retirement? Results from the SHARE survey. Journal of health economics. 2016;45:27-46.
47. Stenholm S, Solovieva S, Viikari-Juntura E, Aalto V, Kivimäki M, Vahtera J. Change in body mass index during transition to statutory retirement: an occupational cohort study. International Journal of Behavioral Nutrition and Physical Activity. 2017;14(1):85.

48. Rosengren A, Smyth A, Rangarajan S, Ramasundarahettige C, Bangdiwala SI, AlHabib KF, et al. Socioeconomic status and risk of cardiovascular disease in 20 low-income, middle-income, and high-income countries: the Prospective Urban Rural Epidemiologic (PURE) study. The Lancet Global Health. 2019;7(6):e748-e60. doi: 10.1016/S2214-109X(19)30045-2.
49. Dégano IR, Marrugat J, Grau M, Salvador-González B, Ramos R, Zamora A, et al. The association between education and cardiovascular disease incidence is mediated by hypertension, diabetes, and body mass index. Scientific reports. 2017;7(1):12370-. doi: 10.1038/s41598-017-10775-3. PubMed PMID: 28959022.
50. Schultz William M, Kelli Heval M, Lisko John C, Varghese T, Shen J, Sandesara P, et al. Socioeconomic Status and Cardiovascular Outcomes. Circulation. 2018;137(20):2166-78. doi: 10.1161/CIRCULATIONAHA.117.029652.
51. Sillars A, Celis-Morales CA, Ho FK, Petermann F, Welsh P, Iliodromiti S, et al. Association of Fitness and Grip Strength With Heart Failure: Findings From the UK Biobank Population-Based Study. Mayo Clinic Proceedings. 2019;94(11):2230-40. doi: 10.1016/j.mayocp.2019.04.041.
52. Lee M-R, Jung SM, Kim HS, Kim YB. Association of muscle strength with cardiovascular risk in Korean adults: Findings from the Korea National Health and Nutrition Examination Survey (KNHANES) VI to VII (2014–2016). Medicine (Baltimore). 2018;97(47).
53. Rein P, Saely C, Vonbank A, Zanolin D, Drexel H. Hand grip strength significantly predicts cardiovascular event risk in patients with type 2 diabetes. J Am Coll Cardiol. 2016;67(13 Supplement):2029. doi: 10.1016/S0735-1097(16)32030-7.
54. Tikkanen E, Gustafsson S, Ingelsson E. Associations of Fitness, Physical Activity, Strength, and Genetic Risk With Cardiovascular Disease. Circulation. 2018;137(24):2583-91. doi: 10.1161/CIRCULATIONAHA.117.032432.
55. Ruan Y, Guo Y, Zheng Y, Huang Z, Sun S, Kowal P, et al. Cardiovascular disease (CVD) and associated risk factors among older adults in six low-and middle-income countries: results from SAGE Wave 1. BMC public health. 2018;18(1):778. doi: 10.1186/s12889-018-5653-9.
56. Yusuf S, Hawken S, Ôunpuu S, Dans T, Avezum A, Lanas F, et al. Effect of potentially modifiable risk factors associated with myocardial infarction in 52 countries (the INTERHEART study): case-control study. The lancet. 2004;364(9438):937-52.
57. Dowd JB, Zajacova A. Does self-rated health mean the same thing across socioeconomic groups? Evidence from biomarker data. Ann Epidemiol. 2010;20(10):743-9. doi: 10.1016/j.annepidem.2010.06.007. PubMed PMID: 20816313.

Supporting information

S1 Annex. Supplementary materials. The supplementary material contains detailed information on comparison of included and excluded participants, behavioral risks overtime, sensitivity analysis of multiple imputation and complete case analysis, checking for imputation model fit, comparison of models with various correlation structure, and stratified analysis for Belgium and France~~, and the mediation analysis~~.

Formatted: Heading 1, Left, Space After: 12 pt, Line spacing: single

Formatted: Space Before: 12 pt

Formatted: TableTitle, Line spacing: Double

Response to Reviewers

PONE-D-20-21259

Socioeconomic and behavioral determinants of cardiovascular diseases among older adults in Belgium and France: a longitudinal analysis from the SHARE study

PLOS ONE

First of all, I wish to thank the editor and the reviewers, also on behalf of all authors, for their valuable and constructive comments to improve our manuscript. We revised the manuscript based on the comments and issues raised by the editor and the reviewers. We are confident that we have addressed all these comments adequately and we therefore hope that the manuscript is accepted for publication. Find hereunder a point by point reply to the comments and questions raised by the editor and the two reviewers.

Editor

1. Please ensure that your manuscript meets PLOS ONE's style requirements, including those for file naming.

Response: Thank you for your suggestions. We have reviewed the manuscript to meet the journal requirements and we hope that the manuscript in its current form does so.

2. Please provide additional details regarding participant consent. In the ethics statement in the Methods and online submission information, please ensure that you have specified (1) whether consent was informed and (2) what type you obtained (for instance, written or verbal, and if verbal, how it was documented and witnessed). If your study included minors, state whether you obtained consent from parents or guardians. If the need for consent was waived by the ethics committee, please include this information.

Response: Thank you for your suggestions. We used publicly available data (available via the website http://www.share-project.org/data-documentation/data-documentation-tool.html) for a secondary data analysis. In order to clarify this, we have added the following sentences in line 208 - 214 of the revised version of the manuscript:

"We used publicly available data (available via the website http://www.share-project.org/data-documentation/data-documentation-tool.html) for a secondary data analysis. SHARE underwent a review of ethical standards by the University of Mannheim's internal review board (IRB). Ethical considerations including written informed consent has been taken care of by another institution. Details on the conduct of the study including the ethical approval can be found elsewhere (Alcser, Benson et al. 2005)."

Reviewer # 1

1. The work is very well conducted, the ideas are original and relevant. The results are very interesting and will be very beneficial for the scientific community. Additionally, I find it well written and easy to understand. I would just like to comment that in table 1 in the Sex (Female) variable, I noticed that there is a typo: you need to close the parentheses.

Response: Thank you for your feedback and appreciation for our work. We corrected the typo accordingly.

Reviewer # 2

1. The authors conducted a large cohort study evaluating socioeconomic determinants and cardiovascular disease using data collected from several waves of the SHARE study. By using marginal GEE models the investigators were able to account for between- and within- subject effects of social determinants. The paper was well thought out in many ways but there are some areas for improvement. Suggested edits/comments could be found below.

Response: Thank you for your feedback. The comments were very helpful and we revised the manuscript thoroughly. We hope that we addressed all your concerns in the revised manuscript.

2. Please provide some analysis of individuals for which the outcomes of interest were missing. The authors state in line 108-109 that only persons with outcome data were contained in the sample but it is not clear how not including these persons may have biased the results. Even some descriptive analyses among persons with missing outcome data would be helpful. This is exceptionally important for the outcome which may be missing a large proportion of individuals who are deceased and therefore were not included in the sample.

Response: Thank you for your feedback. As we mentioned in line 108 to 110, we excluded individuals who are aged < 50 years, individuals that already have CVD at enrollment and those that did not have information on the CVD outcome variables at enrollment. Participants with intermediate missingness in terms of the outcome variables are included in the analysis and managed using the MI technique. The main reason to exclude individuals with no CVD outcome information at enrollment is to make sure that the individuals that are included are free from CVD events prior to study initiation. In total, 467 participants were excluded (108 being aged below 50 years, 117 with missing outcome values at enrollment and 242 individuals that already had a history of CVD at enrollment). Although we assume missingness of CVD outcomes at enrollment to be completely at random (MCAR) by not including those 117 participants, the percentage of missing outcome data is below 0.8% and we therefore believe that the impact on the inference is limited when the underlying missingness mechanism is mis-specified. Nevertheless, we do agree with the reviewer that a descriptive analysis to deal with the baseline missingness could be explored. As recommended by the reviewer, we performed a descriptive analysis to compare the included individuals and the individuals that were excluded due to missing outcome values (n=117) in terms of a set of covariates. The descriptive

results thereof are presented in table 1 of the supplementary material. The results show that individuals who were included are comparable with the excluded ones in terms of socioeconomic characteristics. We also added a description in line 166 to 171 of the revised manuscript.

3. Related to this point, proportions of missingness in tables 3-4 were helpful, however, in general it would be helpful to know more about the multiple imputations models. How were variables selected and were there auxiliary variables used to specify the missing model?

Response: Thank you for this comment. Indeed, we used auxiliary variables in combination with the covariate information used in the substantive model(s) in order to specify the imputation models. Examples of such auxiliary variables include family size, activities of daily living, limitation of activities, self-perceived health, among other variables. The perspective taken in the Multiple Imputation by Chained Equations (MICE) approach, also referred to as full conditional specification, is one of a specification of several imputation models which sequentially imputed missing values for a given variable when regressed against all other covariates available. For more details regarding this approach and the justification of full conditional specification, we refer the reader to (Van Buuren 2018). We revised the description in line 194 to 200 of the analysis section in order to clarify this. Moreover, more details concerning the imputation models and the covariates included therein are included in page 6 of the supplementary material. In general, the inclusion of auxiliary variables in the imputation models, next to the covariates present in the substantive model(s), albeit potentially unimportant from an explanatory perspective, leads to a more precision in terms of estimation of the model parameters in the final substantive model.

4. Please clarify if any of the "multivariate". This appears to be used interchangeably but it seems as though "multivariable" may be the correct wording. The different types of models have very different implications.

Response: We agree with the reviewer that a multivariate analysis is different from a multiple or multivariable one, the latter being the analysis that has been conducted in our manuscript. Therefore, we revised the manuscript to consistently use ‘multivariable’ throughout the manuscript.

5. While the authors allude to some of the limitations of using self-reported data, it might be helpful to include some more discussion of how measurement error may have biased some of these findings (e.g., low SES underreporting certain social behaviors such as alcohol/tobacco use/abuse, how does self-reporting CV events limit this analysis?).

Response: We kindly agree with the reviewer that those in low socioeconomic status usually have a higher likelihood of under-reporting CVD status, risks and other health related issues, which might nullify the effect size of SES on CVD. This indicates the effect of SES on CVD could be even more than what we found. We elaborated more extensively on the limitation of the self-reported data and discussed this in view of the reviewer’s comment in line 367 to 374 of the revised manuscript.

6. The mediation analysis was well thought out but since this contains so many similar social constructs, the authors may benefit from removing it from the main text and supplement. It can be explored in future work when developing a causal model rather than the present predictive one. Collinearity also may be an issue between some of the crudely defined socioeconomic factors.

Response: We agree that the mediation analysis needs more exploration. We accept that this study and more importantly its design did not fully comply with nor allow for a formal and in-depth mediation analysis as we have a number of related determinants and mediators. Separate mediation analyses are needed, principally for each determinant-mediator-outcome relationship. We will consider to perform an extensive mediation analysis in the continuing analysis. Initially, we believed that this mediation analysis would provide some indications for future studies. However, to avoid confusion and mis-understanding for readers, we removed it from the main text and the supplement.

7. A similar analysis looking at incident CVD cases would be interesting either for this study or in the future. Is there a way to exclude persons with prevalent CVD events from earlier waves and at baseline of this study to understand if one of these factors (i.e., retirement status, depression) cause CVD in this population?

Response: Thank you for your feedback. In fact, we excluded those individuals with CVD during the first wave of their enrollment as we mentioned in line 108 to 110 of the methods section. However, in this study, as the survey is every two years, we could not ascertain the exact number of CVD events (MI, stroke, etc..) and the exact date of such an event in between the waves. This made the incidence analysis somewhat complicated. Nevertheless, we do believe that this is very interesting and a potential avenue for further research. As suggested, we will consider to perform incidence analysis in the future.

8. Reporting variability within and between countries is an advantage of using hierarchical models such as the one employed in this study but there is no mention of it in the text. Only descriptive results were presented between the countries. Please include more information on how these countries differ.

Response: Thank you for your comment. We explored the difference between two countries extensively in the revised version. Besides the descriptive statistics, we also compared the difference between countries in several ways. Firstly, we compared both countries using differences in socioeconomic as well as the outcome variable. Secondly, as we have mentioned in line 187 to 190, we developed a separate GEE model for the two countries to assess whether the determinants vary across countries. However, the results showed no significant difference in the effect sizes of determinants between countries (detailed results are indicated in table 7 and 8 of the supplementary material). Thirdly, we performed a combined analysis without the inclusion of country-specific determinants under the assumption that such differences were unimportant based on our earlier stratified analysis. Hence, we finally preferred to present the results of the (combined) GEE model, for both countries combined.

9. It will be generally important to state whether this is a predictive model that identifies risk factors and not a causal model which may be limited to confounding and selection biases.

Response: Thank you for your feedback. We do agree that the aim of this study is to identify risk factors for the occurrence of CVD, i.e. a so-called risk factor identification study. We stated this explicitly in the discussion section and indicated that our model is not a causal one, rather one that focuses on association and not on (the establishment of) causality. We believe that this study would provide overall insight for further individual causal study.

References

Alcser, K. H., G. Benson, A. Börsch-Supan, A. Brugiavini, D. Christelis, E. Croda, M. Das, G. de Luca, J. Harkness and P. Hesselius (2005). "The survey of health, aging, and retirement in Europe—Methodology." Mannheim Mannheim Research Institute for the Economics of Aging (MEA).

Van Buuren, S. (2018). Flexible imputation of missing data, CRC press.

Hamid Y. Hassen

Hamid.hassen@uantwerpen.be
